# Configurational Isomerism in Bimetallic Decametalates

**DOI:** 10.3390/ma17143624

**Published:** 2024-07-22

**Authors:** Aleksandar Kondinski

**Affiliations:** Department of Chemical Engineering and Biotechnology, University of Cambridge, Philippa Fawcett Drive, Cambridge CB3 0AS, UK; aleksandar@kondinski.com

**Keywords:** polyoxometalates, density functional theory, isomerism, metal–metal bonds

## Abstract

In this work, we report on the development of a computational algorithm that explores the configurational isomer space of bimetallic decametalates with general formula ${\left[M_{x}M_{10-x}^{\prime }O_{28}\right]}^{q}$. For *x* being a natural number in the range of 0 to 10, the algorithm identifies 318 unique configurational isomers. The algorithm is used to generate mixed molybdenum(VI)–vanadium(V) systems ${\left[{\mathrm{Mo}}_{x}V_{10-x}O_{28}\right]}^{8-}$ for x=0,1,2, and 3 that are of experimental relevance. The application of the density functional theory (DFT) effectively predicts stability trends that correspond well with empirical observations. In dimolybdenum-substituted decavanadate systems, we discover that a two-electron reduction preferentially stabilizes a configurational isomer due to the formation of metal–metal bonding. The particular polyoxometalate structure is of interest for further experimental studies.

## 1. Introduction

Polyoxometalates (POMs) are a class of metal–oxo clusters that play significant roles in catalysis [1], life sciences [2,3], and nanoelectronics [4,5]. These clusters primarily consist of early transition metals (V, Nb, Ta, Mo, W), though compositions with late transition metals and actinide elements (Cu, Ni, Pd, Pt, Au, U, Np) are also common [6,7]. POMs exhibit a high degree of structural diversity, with few key structural archetypes such as Keggin [8], Lindqvist [9], and Anderson–Evans [10], serving as foundations for the development of new, functional materials through crystal [11,12] and molecular engineering [13,14,15].

The gradual formal substitution of primary POM archetype with heterometals or heterogroups has been traditionally used as a design principle for the development of new functional materials [16,17,18]. This approach has been broadly applied in the development of substituted Kegginoids with enhanced catalytic [19,20] and magnetic response [21]. However, the formal substitution within POMs creates a requirement for the proper description of their configurational space described by all unique and individual isomers [22,23,24,25]. Theoretical insights into the relative stability and electronic properties of isomeric POMs have been frequently studied over the past two decades [18,26]; however, owing to the computational cost, many studies have frequently focused on small sets of configurational isomers [18]. However, more recent approaches, combining configurational space exploring algorithms, DFT computations, and empirical insights enable the rapid navigation of that space, yielding valuable insights into the complex archetype dynamics of POMs as a function of building block availability [27].

**Figure 1:**
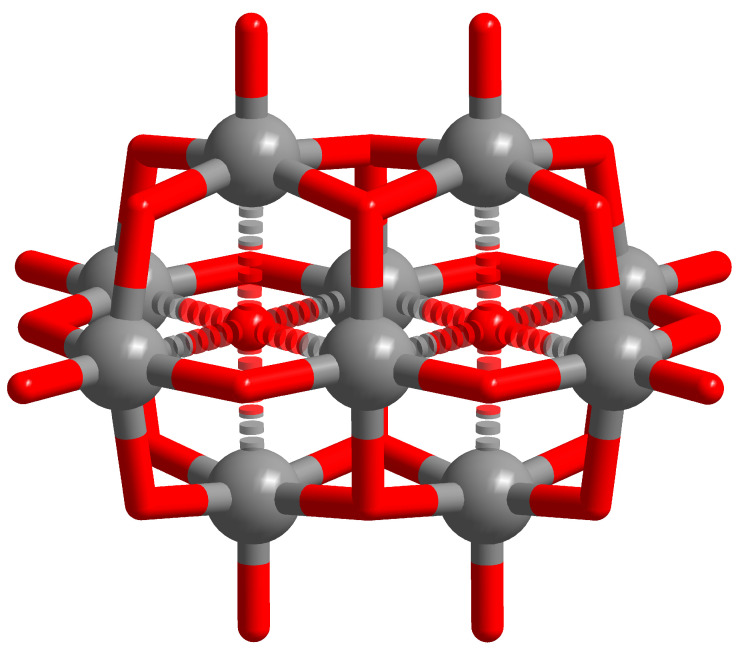
Ball and stick representation of a generic decametalate ${\left[M_{10}O_{28}\right]}^{n-}$ structure. Color code: M = gray, O = red.

The decametalate structure represents an important structural archetype in POM chemistry (see Figure 1). In the latter structure, ten metal centers occupy the vertices of two virtual octahedra with single-edge sharing topology [17]. Eight of these metal centers connect to terminal oxo atoms. All atoms interconnect to one another *via* $\mu_{2}$, $\mu_{3}$, and $\mu_{6}$ oxo ligands. Two metal centers, forming the core of the POM, do not exhibit terminal oxo ligands. The decavanadate ${\left[V_{10}^{V}O_{28}\right]}^{6-}$ is the most representative and studied decametalate, showing a broad set of applications in the realm of POM chemistry [9,28,29]. Nb^*V*^- and Ta^*V*^-based analogs of the decavanadate are also known, and they have been extensively studied over the past years [30,31,32,33].

**Figure 2:**
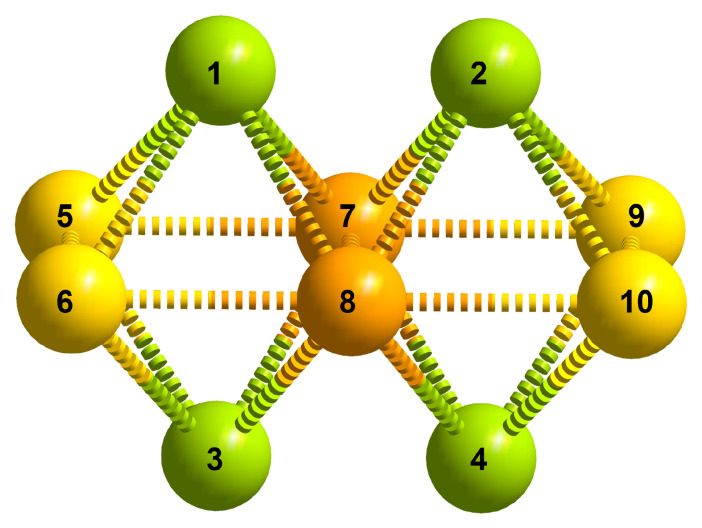
Representation of the relative neighboring positions between the ten M centers in a generic ${\left[M_{10}O_{28}\right]}^{n-}$. Green, yellow, and orange coloring is used to depict atom centers that hold configurationally equivalent positions.

In terms of single-addenda metal substitution of the decametalate archetype, there are a few structure reported in the literature, namely ${\left[H_{2}{\mathrm{Pt}}^{\mathrm{IV}}V_{9}O_{28}\right]}^{5-}$, ${\left[H_{2}{\mathrm{Fe}}^{\mathrm{III}}{\mathrm{Nb}}_{9}O_{28}\right]}^{6-}$, ${\left[H_{2}{\mathrm{Ni}}^{\mathrm{II}}{\mathrm{Nb}}_{9}O_{28}\right]}^{6-}$, ${\left[{\mathrm{Ti}}^{\mathrm{IV}}{\mathrm{Nb}}_{9}O_{28}\right]}^{7-}$, ${\left[{\mathrm{HTe}}^{\mathrm{VI}}V_{9}O_{28}\right]}^{4-}$, and ${\left[I^{\mathrm{VII}}V_{9}O_{28}\right]}^{4-}$ [34,35,36,37,38,39]. In each of the single-metal-substituted systems, position 7 or 8 (see Figure 2) has been formally substituted. In contrast, single-Mo^VI^ substitution leads to $\left[H_{2}{\mathrm{MoV}}_{9}O_{28}\right]$, in which Mo^VI^ occupies the peripheral sites (i.e., 1, 2, 3, or 4, as shown in Figure 2). Two-metal substitution is also noted for Mo^VI^ substitution, leading to the two different [1,2] (*syn*) and [1,4] (*anti*) isomers of ${\left[{\mathrm{Mo}}_{2}V_{8}O_{28}\right]}^{4-}$, as differentiated based on NMR spectroscopy [40,41]. The isomer arrangement is also noted for Ti^IV^-substituted polyoxoniobate clusters ${\left[{\mathrm{Nb}}_{8}{\mathrm{Ti}}_{2}O_{28}\right]}^{8-}$ [37,42]. Triaddenda metal substitutions are noted for ${\left[{\mathrm{Mo}}_{3}V_{7}O_{28}\right]}^{3-}$ obtained through microwave synthesis [40].

To provide insights into the configurational space of bimetallic decametalates, in this work, we first develop an algorithm that generates and enumerates the possible structures. Considering the significance of the isomer problem with Mo^VI^ substitution in polyoxovanadate structures, we investigated the electronic structure and stability of ${\left[{\mathrm{Mo}}_{x}V_{10-x}O_{28}\right]}^{q}$ for x=0,1,2, and 3. Once relative isomer stability is established, we narrow into the electronic structure and properties of [1,2] (*syn*) and [1,4] (*anti*) isomers ${\left[{\mathrm{Mo}}_{2}V_{8}O_{28}\right]}^{4-}$ and their two-electron-reduced species.

## 2. Materials and Methods

Numeric labels of the atomic positions within the decametalate structure are designated in Figure 2. The positions are color-coded green, yellow, and orange—corresponding to positions typically denoted as A, B, and C, respectively. This categorization aids in describing the local environments of the metal centers. The equivalence of these positions can be formulated as 1↔2↔3↔4 for Position A (green), 5↔6↔9↔10 for Position B (yellow), and 7↔8 for Position C (orange). Utilizing this established equivalence, an algorithm was developed to explore the configurational space of the substituted system more effectively (Section 2.1). The labeling of positions A, B, and C is also used for qualitative assessments in the Results Section.

### 2.1. Decametalate Isomerizer

The algorithm generates unique bimetallic decametalate configomers (i.e., configurational isomers) by examining all possible arrangements of relative atomistic positions in a 3D space, taking into account rotational symmetries along the x, y, and z axes. Each configuration is considered unique if it cannot be transformed into another configuration by these rotations, representing an orbit under the action of the spatial orthogonal group in three dimensions (SO(3)). First, we define a set of positions B={1,2,…,n}, where *n* is the number of atomistic positions. A configuration $C_{k}$ is a subset of *B* with *k* elements, where 0≤k≤n. We define rotation mappings $\sigma_{y}$, $\sigma_{x}$, and $\sigma_{z}$ for the y-axis, x-axis, and z-axis, respectively, to model the transformations of SO(3). These mappings specify how the atomistic positions change under each rotation. To determine if a configuration is unique, we generate all possible rotations of $C_{k}$ and check if any of these rotations match previously seen configurations. This is performed by applying each rotation function to $C_{k}$ and comparing the results. If a configuration or any of its rotations are not already in the set of unique configurations, it is added to this set. The algorithm iterates through each possible number of atom-substituted positions from 0 to *n*, generating all configurations for each *k*. It then filters out nonunique configurations by checking against the set of previously seen configurations. Finally, it accumulates the total number of unique configurations across all steps, providing the total number of unique isomers for the given set of positions (see Algorithm 1). An expansion of the algorithm that includes the possible mirror images enables further sorting of enantiomeric pairs within the sets of configurational isomers.
**Algorithm 1:** Decametalate Isomerizer  **Input**: Number of positions *n*  **Output**: Set of all unique configurations T 
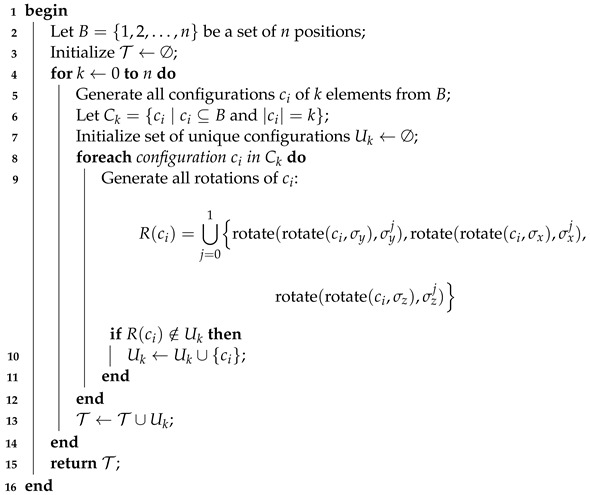


### 2.2. Molecular DFT Calculations

The DFT calculations were performed using the Amsterdam density functional code (ADF2022) [43,44]. We used Slater basis sets of triple-zeta quality with one polarization function (TZP) [45]. Scalar relativistic corrections were considered within the zero-order regular approximation (ZORA) [46]. Solvent effects were modeled using the conductor-like screening model (COSMO) [47,48], with the parameters for water (ϵ = 78.39; solvent radius = 1.93 Å). The computational screening of all positional bare and protonated isomers of ${\left[{\mathrm{Mo}}_{x}V_{10-x}O_{28}\right]}^{n-}$ was performed using the GGA Becke exchange plus the Perdew 86 correlation (BP) functional (i.e., the BP86/ZORA-scalar/TZP/COSMO level) [49,50]. To obtain more accurate information on the gap energy and charge distributions, for selected structures (see Section 2.1), we performed single-point calculations also at the B3LYP/ZORA-scalar/TZP/COSMO level. For the two-electron-reduced species ${\left[{\mathrm{Mo}}_{2}V_{8}O_{28}\right]}^{6-}$, unrestricted spin polarized calculations were performed for the triplet state. Optimization was maintained at the BP86/ZORA-scalar/TZP/COSMO level, with single-point calculations executed at the B3LYP/ZORA-scalar/TZP/COSMO level.

## 3. Results

### 3.1. Total Number of Isomers

The results presented in Table 1 were generated using the algorithm described in the methodology (Section 2), which examines all possible arrangements of relative atomistic positions in 3D space and accounts for rotational symmetries. As the level of substitution *x* increases, the number of unique isomers initially increases, reaching a maximum at x=5, and then decreases symmetrically. This trend reflects the increasing complexity and combinatorial possibilities up to the midpoint of substitution, followed by a reduction in the number of unique configurations as the system becomes more constrained toward full substitution. The total structural space covered by this substitution is the sum of the number of unique isomers for each level of substitution, which amounts to 318 unique structures. The consideration of mirror images of individual isomers also provided the possibility to enumerate the enantiomeric pairs that show a maximum of 21 enantiomeric pairs for x=5. Lists of the calculated unique configurational isomers are provided as part of the Supporting Information.

### 3.2. Structure, Relative Stability, and Electronic Properties

In the decametalate structure, all ten metal centers are in an octahedral environment, albeit with deviations from the ideal. The calculated bond ranges (see Supporting Information) for the complete decavanadate topology show V-O bond lengths within expected ranges for terminal V-O (1.6331–1.6370 Å), V to $\mu_{2}$-O (1.7206–2.0332 Å), V to $\mu_{3}$-O (1.9357–2.0230 Å), and V to $\mu_{6}$-O (2.0957–2.3793 Å). The V atoms at the A, B, and C sites may have different connectivity to the bridging oxo ligands, giving rise to slightly wider ranges in the bonding between V to $\mu_{2}$-O and V to $\mu_{6}$-O atoms. All calculated bond lengths are comparable to other DFT studies on these clusters, as reported in the literature [51]. The introduction of a single Mo^6+^ atom at positions 1, 5, and 7 in the monosubstituted ${\left[{\mathrm{MoV}}_{9}O_{28}\right]}^{5-}$ primarily affects the distances of the remaining V-O bonds towards the central $\mu_{6}$-O. This discrepancy is most significant at position 5, altering the overall V-O range from 2.0957–2.3793 Å in the starting decavanadate to 2.0025–2.4781Å in the substituted cluster. The presence of two Mo centers, as in [5,6]-${\left[{\mathrm{Mo}}_{2}V_{8}O_{28}\right]}^{4-}$, does not further impact the central $\mu_{6}$-O (remaining V-O ranges at 2.0194–2.4930Å), although this effect is slightly more pronounced in the trisubstituted [5,7,8]-${\left[{\mathrm{Mo}}_{3}V_{7}O_{28}\right]}^{3-}$, where the range extends to 2.2890–2.5703 Å.

**Figure 3:**
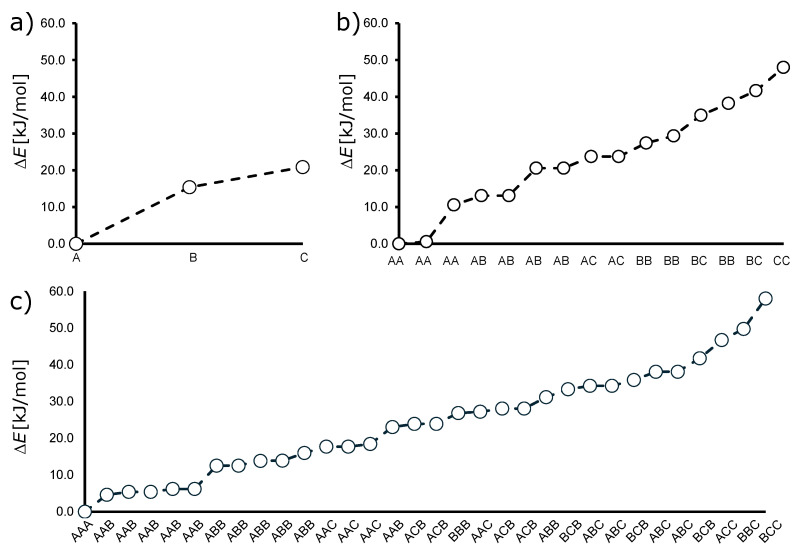
Relative bonding energies (ΔE) for different configurational isomers calculated at the SP/B3LYP/TZP/COSMO-water level of theory. Configurational isomers of (**a**) ${\left[MoV_{9}O_{28}\right]}^{5-}$; (**b**) ${\left[Mo_{2}V_{8}O_{28}\right]}^{4-}$; (**c**) configurational isomers of ${\left[Mo_{3}V_{7}O_{28}\right]}^{3-}$. Isomers are ordered in terms of decreasing bonding energy and designated through A, B, and C Mo-occupied positions. For more details, see the Supporting Information file.

Relative bonding energies in polyoxovanadates, even of 50 kJ/mol, have not been sufficient to dismiss some structures as less likely to form than others [18]. In fact, in experiments, structures calculated within this energy range have been successfully produced [22,24]. Keeping this in mind, we calculated the energy differences when occupation occurs over sites 1, 5, and 7 (i.e., A, B, and C) for ${\left[{\mathrm{MoV}}_{9}O_{28}\right]}^{5-}$. As shown in Figure 3a, the highest relative stability is observed for the occupation of position 1 (i.e., A-type metal center). Next comes the occupation of site B (i.e., position 5) at 15.39 kJ/mol higher energy, which is likely enthalpy driven. Occupation of position 7 results in an energy increase of 20.85 kJ/mol. Although the latter substituted position does not necessarily create higher disturbance to the oxo bridging to the rest of the vanadate structure, it is important to note that Mo^6+^ is effectively sharing oxygen atoms with all remaining V^5+^ centers. These qualitative differences in formal charge configuration can lead to destabilization; however, it is not strongly evident from the overall energy that the formation of such configomer is significantly less competitive in comparison with the other. In fact, the isolation of [IV_9_O_28_]^4−^, which holds a higher formal charge on I^7+^, shows that centers with even more positive charges can be accommodated at site 7 (i.e., C-type position); thus, appropriate conditions of substitution for the isolation of position 7 substituted ${\left[{\mathrm{MoV}}_{9}O_{28}\right]}^{5-}$ may not have been found yet.

In ${\left[{\mathrm{Mo}}_{2}V_{8}O_{28}\right]}^{4-}$, shown in Figure 3b, the configuration with substitutions at positions [1,4] is the most stable, showing the lowest energy, and thus is taken as a base for comparison. This structure is followed by favorable [1,2] configuration, which is only 0.59 kJ/mol higher in energy. As discussed in the introduction, this may not be surprising as both configurations have been isolated in experiments. The next stable configuration, [1,3], is 10.58 kJ/mol higher in energy than [1,4], indicating a trend where stability decreases as substitutions shift from A to B and then to C positions, culminating in the [7,8] configuration, which is 47.96 kJ/mol higher in energy. For the three substituted systems shown in Figure 3c, the most stable configuration is [1,2,3], with all Mo atoms positioned at A, reflecting the highest stability. This is then followed by [1,3,8] and the mirror isomers [1,4,6], and [1,4,5] follow closely, showing minor energy differences of about 4.63 kJ/mol and 5.40 kJ/mol, respectively. The least stable configuration in this series is [5,7,8], which exhibits a significant increase in relative bonding energy of 57.99 kJ/mol, underscoring the impact of substituent positioning on molecular stability.

In fully oxidized polyoxometalates (POMs), the highest occupied molecular orbitals (HOMOs) are primarily composed of oxygen-centered p-type orbitals. While these can conceptually resemble the ‘oxo band’ seen in bulk metal oxides, it is crucial to recognize that in POMs, they represent discrete molecular orbitals rather than continuous bands [18,26]. Conversely, the lowest unoccupied molecular orbitals (LUMOs) are predominantly localized over the metal centers, drawing a parallel to the `metal band’ in bulk materials. This framework facilitates understanding of the typical ligand-to-metal charge transfer upon reduction, which populates the metal centers and modifies the electronic structure of the POMs [18,26].

**Figure 4:**
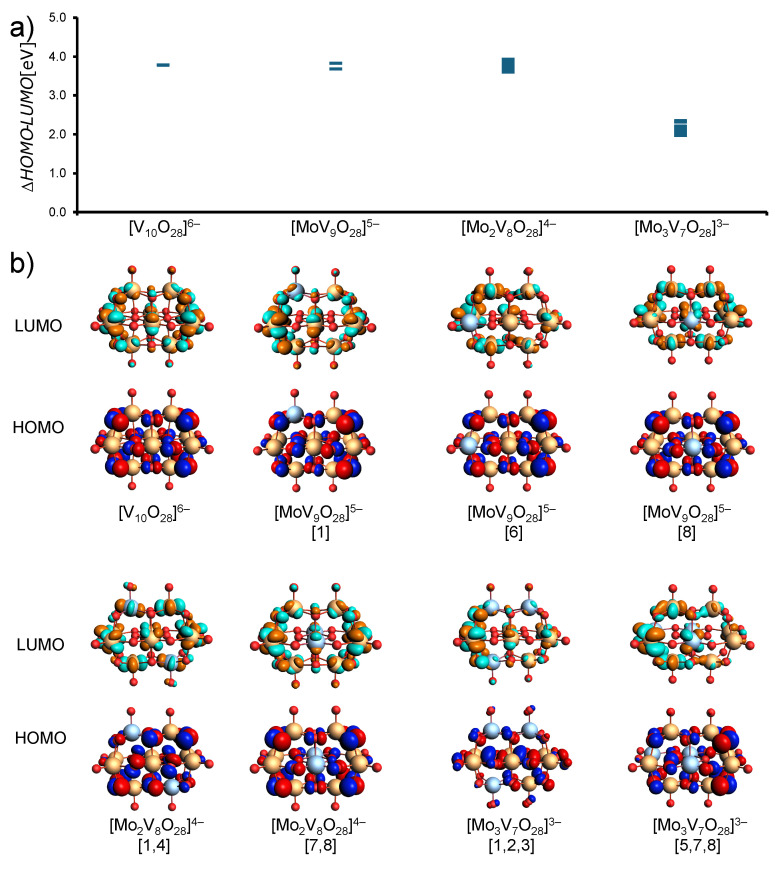
(**a**) HOMO–LUMO gap energies for various polyoxometalate compounds: ${\left[V_{10}O_{28}\right]}^{6-}$, ${\left[MoV_{9}O_{28}\right]}^{5-}$, ${\left[Mo_{2}V_{8}O_{28}\right]}^{4-}$, and ${\left[Mo_{3}V_{7}O_{28}\right]}^{3-}$. (**b**) Calculated HOMO (bottom row) and LUMO (top row) molecular orbitals for the same compounds at the SP/B3LYP/TZP/COSMO-water level of theory. Specific configurations: ${\left[V_{10}O_{28}\right]}^{6-}$, ${\left[MoV_{9}O_{28}\right]}^{5-}$ [1], [6], [8], ${\left[Mo_{2}V_{8}O_{28}\right]}^{4-}$ [1,4], [7,8], and ${\left[Mo_{3}V_{7}O_{28}\right]}^{3-}$ [1,2,3], [5,7,8]. color-coded orbitals illustrate electron density distribution in the HOMO and LUMO.

However, the relative energies and population preference will be dependent on the structure and its composition (see Figure 4a). In the case of one-metal substitutions, we observe that HOMO energies are relatively stable and in the range of −7.18 to −7.16 eV for substitution at positions A, B, and C; however, the calculated LUMO energies appear to be a little bit more sensitive showing −3.35 eV for positions A and C to −3.47 eV for position B. The HOMO–LUMO gaps are consistent for positions A and C at 3.83 eV, with a slight decrease to 3.68 eV for position B. This gap energy is in the range of the calculated gap energy of the decavanadate 3.78 eV, which suggests that single-${\mathrm{Mo}}^{6+}$ substitution lowers the energies of both the HOMO and LUMO, however, without significantly affecting the gap energy. All of the single-substituted decavanadates have dipoles that differ in strength.

In the case of two two-metal substitutions, HOMO energies range more broadly from −7.67 to −7.53 eV, while the LUMO energies also exhibit a broader range, which makes the HOMO–LUMO gaps fluctuate between 3.93 and 3.60 eV (see Figure 4a,b). Interestingly, the gap energies calculated for the most stable isomers covering syn and anti-Mo substitution at 1,2 and 1,4 have almost identical gap energies of 3.92 and 3.93 eV. Compared with the decavanadate system, the 1,2- and 1,4-Mo-substituted systems exhibit higher HOMO–LUMO gap energies, making them harder to reduce. Other species such as [5,10] and the least stable [7,8] have gap energies of 3.79 eV and 3.87 eV, respectively. The configurations [1,4], [5,10], and [7,8] have no net dipole moments, while the remaining ones all show some form of dipole polarization due to the different arrangements of the Mo centers.

The substitution of three centers introduces an uneven number of substitutions, which not only leads to higher overall charges within the cluster but also disrupts the LUMO (see Figure 4b). Specifically, this disruption manifests as a preference for localizing the LUMO over the vanadium (V) centers of the cluster. Such localization suggests that certain sections of the cluster, particularly those around vanadium centers, are more susceptible to reduction compared with others. In conjunction with the Mo centers, this uneven localization contributes to a pronounced reduction in the HOMO–LUMO gap energies. The altered electronic landscape in these trisubstituted systems results in HOMO energies ranging from −7.09 to −6.86 eV and LUMO energies between −4.99 and −4.74 eV. Consequently, the calculated HOMO–LUMO gaps in these systems vary from approximately 1.97 to 2.35 eV.

DFT calculations of relative energies for [1,2,3], [1,3,9], [1,4,6], and [1,2,6] isomers of ${\left[Mo_{3}V_{7}O_{28}\right]}^{3-}$ have been previously performed [40]. While these findings align with expected stability orders, they cover only a subset of the 32 potential isomers. This study expands upon prior work by conducting a comprehensive analysis of all isomers generated through our isomerizer algorithm, thus providing a more complete understanding of the configurational space and stability trends within these polyoxometalate structures.

### 3.3. Two-Electron Reduction and Metal–Metal Bonding

Over the past few years, increased interest has been directed to the study of highly reduced POMs and the evolution of metal–metal bonding in some of these highly reduced systems [4,52,53]. This interest further motivated the examination of the structural and electronic properties of some selected bimetallic decametalate species. The configurations ${\left[Mo_{2}V_{8}O_{28}\right]}^{4-}$ [1,4] and ${\left[Mo_{2}V_{8}O_{28}\right]}^{4-}$ [1,2], previously reported as anti- and syn-isomers, respectively [40,41], were chosen for further theoretical investigation, as they are synthetically accessible and therefore can be of interest for further experimental investigations.

By adding an additional pair of electrons, it was observed that in the ${\left[Mo_{2}V_{8}O_{28}\right]}^{6-}$ [1,4] configuration, the two Mo centers are too far apart to facilitate bonding through the overlap of $d_{xy}$ orbitals. This spatial arrangement precludes the formation of a metal–metal bond in this configuration. Conversely, the ${\left[Mo_{2}V_{8}O_{28}\right]}^{4-}$ [1,2] configuration provides a conducive environment for bonding. We explored both a spin-polarized scenario with two unpaired, parallel-spin electrons and a singlet state with paired electrons (closed-shell configuration). Upon optimization, it was revealed that in the spin-polarized (triplet) scenario, the two Mo centers remain significantly apart at 3.2771 Å. However, when a singlet state is formed, the interatomic distance contracts significantly to 2.7573 Å, indicating substantial contraction and suggesting bonding supported by the exact overlap of the $d_{xy}$ orbitals.

**Figure 5:**
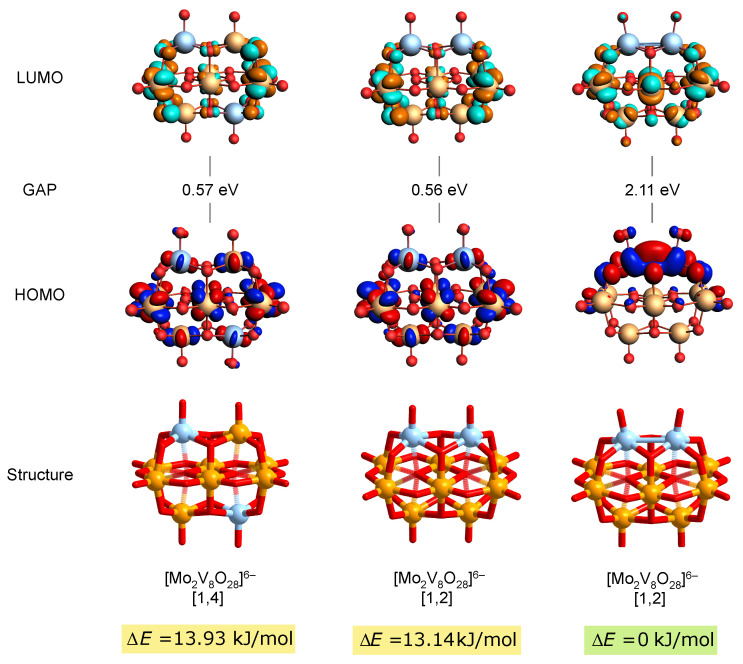
Electronic and structural properties of ${\left[Mo_{2}V_{8}O_{28}\right]}^{6-}$ configurations calculated at the SP/B3LYP/TZP/COSMO-water level of theory. Top row: LUMO molecular orbitals. Second row: HOMO–LUMO gap (GAP) values in eV. Third row: HOMO molecular orbitals. Bottom row: Structures of the polyoxometalates with their corresponding relative bonding energies (ΔE). The structures shown are ${\left[Mo_{2}V_{8}O_{28}\right]}^{6-}$ [1,4] (spin-unrestricted), ${\left[Mo_{2}V_{8}O_{28}\right]}^{6-}$ [1,2] (spin-unrestricted), and ${\left[Mo_{2}V_{8}O_{28}\right]}^{6-}$ [1,2] with metal–metal bonding.

Figure 5 clearly shows the HOMO orbital for the ${\left[Mo_{2}V_{8}O_{28}\right]}^{6-}$ [1,2] singlet state, which supports the metal–metal bonding character of this configuration. Although the HOMO–LUMO gap of 2.11 eV for the metal–metal-bonded configuration might appear low compared with fully oxidized POMs, it aligns with properties observed in molybdenum blues, known for their lower HOMO–LUMO gaps due to metal–metal interactions [4]. The spin-polarized systems present even lower HOMO–LUMO gaps of 0.57 eV and 0.56 eV, reflecting their different electron arrangements that enhance electron mobility across multiple metal centers. In comparison with the singlet-state ${\left[Mo_{2}V_{8}O_{28}\right]}^{6-}$ [1,2], the triplet states of configurations [1,4] and [1,2] are higher in energy, at 13.93 kJ/mol and 13.14 kJ/mol, respectively, thus appearing slightly less favorable energetically. This highlights how stability order between isomers can be further affected by their electronic configuration and reduction state, considering that in the fully oxidized form, [1,4]-${\left[Mo_{2}V_{8}O_{28}\right]}^{4-}$ was calculated to be more stable.

Furthermore, mapping the spin density isosurface of the two-electron-reduced systems shows a predominant population of $d_{xy}$-like orbitals on the V centers in spin-polarized scenarios (see Supporting Information), highlighting vanadium as a preferential site for reduction over molybdenum. In the closed-shell configuration of the [1,2]-${\left[Mo_{2}V_{8}O_{28}\right]}^{6-}$, the LUMO is primarily located on the V atoms. This excitation could lead to a destabilization of the bond as electron density is redistributed towards the V atoms, subtly altering the electronic equilibrium and potentially reducing the strength of the Mo-Mo interactions. Such changes allow the structure to enter a transient spin-polarized state, in which Mo atoms are spatially distanced. Over time, this state can revert back to the more stable Mo-Mo-bonded configuration, demonstrating a dynamic interplay between electron excitation and structural stability.

Mo(V)-Mo(V) units in POMs, as studied using DFT by Lang et al. [54] and Rohmer et al. [55], demonstrate that POMs act as multielectron sources that promote reduction via proton-coupled electron transfer (PCET) and exhibit metal–metal bonding networks that can reorganize under specific conditions. The bond lengths and HOMO–LUMO gap energies calculated in this section are consistent with those reported in their works using a comparable level of theory. Thus, the insights of the new calculations motivate further experimental study of the reported complexes, as well as their further evaluations in applications where controlled electron dynamics are beneficial.

## 4. Conclusions

This study has provided insights into the configurational isomer space in bimetallic decametalates, illustrating how metal substitution influences key electronic properties such as HOMO–LUMO gaps, dipole moments, and bonding energies, which in turn affect molecular stability and behavior. Our investigation into molybdenum-substituted vanadates corroborated empirical observations, which on one hand validates the overall approach and on the other helps in developing an understanding of the electronic structure and stability of the experimentally reported systems. Furthermore, theoretical investigations into experimentally reported but not thoroughly examined structures, such as [Mo_2_V_8_O_28_]^4−^ [1,2], reveal that two-electron reduction to form [Mo_2_V_8_O_28_]^6−^ [1,2] results in metal–metal-bonded clusters that are relatively more stable compared with their spin-polarized analogs. This finding points to interesting charge transfer behavior and the potential stability these interactions may offer, thereby motivating further experimental efforts to explore these promising systems.

## Figures and Tables

**Table 1:** Number of unique configomers and enantiomeric pairs for different levels (*x*) of metal addenda substitution in {M’xM_10−*x*_O_28_}.

*x*	0	1	2	3	4	5	6	7	8	9	10
unique configomers	1	3	15	32	60	66	60	32	15	3	1
enantiomeric pairs	0	0	3	9	19	21	19	9	3	0	0

## Data Availability

The code used for generating and enumerating the structures is available on the Digital Chemistry Git repository. It is provided free of charge under the MIT license. One can access the repository at the following URL: https://github.com/digital-chemistry/dec-isomeriser.git (accessed on 21 July 2024 ). All other data, including optimized geometries, are made available as part of the supporting information to this document.

## Supplementary Materials

The following supporting information can be downloaded at https://www.mdpi.com/article/10.3390/ma17143624/s1: SupportingInformation.pdf containing: (a) configomer lists for all x configurations; (b) Figures S1–S6 depicting spin density isosurfaces, and calculated IR spectra of selected structures; (c) Tables S1–S3: DFT Calculations Summary, Geometric Parameters, and Optimized Geometries; Video_S1.mp4: Video abstract summarising the work.

## Abbreviations

The following abbreviations are used in this manuscript:

POMs	Polyoxometalates
TZP	Triple-Zeta Quality with Polarization (basis set in quantum chemistry)
ZORA	Zero-Order Regular Approximation (a relativistic treatment method)
COSMO	Conductor-like Screening Model (a method for including solvent effects in simulations)
BP	Becke exchange plus the Perdew 86 correlation functional (a type of density functional)
B3LYP	Becke, three-parameter, Lee–Yang–Parr (a hybrid functional in density functional theory)
DFT	Density Functional Theory
GGA	Generalized Gradient Approximation (a type of exchange-correlation functional in DFT)
HOMO	Highest Occupied Molecular Orbital
LUMO	Lowest Unoccupied Molecular Orbital
NMR	Nuclear Magnetic Resonance
