# Supplementary material for: Configurational Isomerism in Bimetallic Decametalates
Source: Materials (Basel). 2024 Jul 22;17(14):3624. doi: 10.3390/ma17143624 (PMC11278824; doi:10.3390/ma17143624)
Supplement: Supplementary file 1 [file materials-17-03624-s001.zip › SupportingInformation.pdf]

Configurational Isomerism in Bimetallic Decametalates
(**Supporting Information**)

Aleksandar Kondinski
Department of Chemical Engineering and Biotechnology, University of Cambridge,
Philippa Fawcett Drive, Cambridge CB3 0AS, United Kingdom Correspondance: aleksandar@kondinski.com

1 Configomer lists

x = 0: 1 unique configurations, 0 enantiomeric pairs

[]

x = 1: 3 unique configurations, 0 enantiomeric pairs

[1], [5], [7]

x = 2: 15 unique configurations, 3 enantiomeric pairs

[1, 2], [1, 3], [1, 4], [1, 5], [1, 6], [1, 7], [1, 8], [1, 9], [1, 10], [5, 6], [5, 7], [5, 8], [5, 9], [5, 10], [7, 8]
Enantiomers: [1, 5] <-> [1, 6]; [1, 7] <-> [1, 8]; [1, 9] <-> [1, 10]

x = 3: 32 unique configurations, 9 enantiomeric pairs

[1, 2, 3], [1, 2, 5], [1, 2, 6], [1, 2, 7], [1, 3, 5], [1, 3, 7],[1, 3, 9], [1, 4, 5], [1, 4, 6], [1, 4, 7], [1, 4, 8], [1, 5, 6], [1, 5, 7], [1, 5, 8], [1, 5, 9], [1, 5, 10], [1, 6, 7], [1, 6, 8], [1, 6, 9], [1, 6, 10], [1, 7, 8], [1, 7, 9], [1, 7, 10], [1, 8, 9], [1, 8, 10], [1, 9, 10], [5, 6, 7], [5, 6, 9], [5, 7, 8], [5, 7, 9], [5, 7, 10], [5, 8, 9]
Enantiomers: [1, 2, 5] <-> [1, 2, 6]; [1, 4, 5] <-> [1, 4, 6]; [1, 4, 7] <-> [1, 4, 8]; [1, 5, 7] <-> [1, 6, 8]; [1, 5, 8] <-> [1, 6, 7]; [1, 5, 9] <-> [1, 6, 10]; [1, 5, 10] <-> [1, 6, 9]; [1, 7, 9] <-> [1, 8, 10]; [1, 7, 10] <-> [1, 8, 9]

x = 4: 60 unique configurations, 19 enantiomeric pairs

[1, 2, 3, 4], [1, 2, 3, 5], [1, 2, 3, 6], [1, 2, 3, 7], [1, 2, 3, 8], [1, 2, 3, 9], [1, 2, 3, 10], [1, 2, 5, 6], [1, 2, 5, 7], [1, 2, 5, 8], [1, 2, 5, 9], [1, 2, 5, 10], [1, 2, 6, 7], [1, 2, 6, 8], [1, 2, 6, 9], [1, 2, 7, 8], [1, 3, 5, 6], [1, 3, 5, 7], [1, 3, 5, 8], [1, 3, 5, 9], [1, 3, 5, 10], [1, 3, 7, 8], [1, 3, 7, 9], [1, 3, 7, 10], [1, 3, 9, 10], [1, 4, 5, 6], [1, 4, 5, 7], [1, 4, 5, 8], [1, 4, 5, 9], [1, 4, 5, 10], [1, 4, 6, 7], [1, 4, 6, 8], [1, 4, 6, 10], [1, 4, 7, 8], [1, 5, 6, 7], [1, 5, 6, 8], [1, 5, 6, 9], [1, 5, 6, 10], [1, 5, 7, 8], [1, 5, 7, 9], [1, 5, 7, 10], [1, 5, 8, 9], [1, 5, 8, 10], [1, 5, 9, 10], [1, 6, 7, 8], [1, 6, 7, 9], [1, 6, 7, 10], [1, 6, 8, 9], [1, 6, 8, 10], [1, 6, 9, 10], [1, 7, 8, 9], [1, 7, 8, 10], [1, 7, 9, 10], [1, 8, 9, 10], [5, 6, 7, 8], [5, 6, 7, 9], [5, 6, 7, 10], [5, 6, 9, 10], [5, 7, 8, 9], [5, 7, 8, 10]
Enantiomers: [1, 2, 3, 5] <-> [1, 2, 3, 6]; [1, 2, 3, 7] <-> [1, 2, 3, 8]; [1, 2, 3, 9] <-> [1, 2, 3, 10]; [1, 2, 5, 7] <-> [1, 2, 6, 8]; [1, 2, 5, 8] <-> [1, 2, 6, 7]; [1, 2, 5, 10] <-> [1, 2, 6, 9]; [1, 4, 5, 7] <-> [1, 4, 6, 8]; [1, 4, 5, 8] <-> [1, 4, 6, 7]; [1, 4, 5, 9] <-> [1, 4, 6, 10]; [1, 5, 6, 7] <-> [1, 5, 6, 8]; [1, 5, 6, 9] <-> [1, 5, 6, 10]; [1, 5, 7, 8] <-> [1, 6, 7, 8]; [1, 5, 7, 9] <-> [1, 6, 8, 10]; [1, 5, 7, 10] <-> [1, 6, 8, 9]; [1, 5, 8, 9] <-> [1, 6, 7, 10]; [1, 5, 8, 10] <-> [1, 6, 7, 9]; [1, 5, 9, 10] <-> [1, 6, 9, 10]; [1, 7, 8, 9] <-> [1, 7, 8, 10]; [1, 7, 9, 10] <-> [1, 8, 9, 10]

x = 5: 66 unique configurations, 21 enantiomeric pairs

[1, 2, 3, 4, 5], [1, 2, 3, 4, 7], [1, 2, 3, 5, 6], [1, 2, 3, 5, 7], [1, 2, 3, 5, 8], [1, 2, 3, 5, 9], [1, 2, 3, 5, 10], [1, 2, 3, 6, 7], [1, 2, 3, 6, 8], [1, 2, 3, 6, 9], [1, 2, 3, 6, 10], [1, 2, 3, 7, 8], [1, 2, 3, 7, 9], [1, 2, 3, 7, 10], [1, 2, 3, 8, 9], [1, 2, 3, 8, 10], [1, 2, 3, 9, 10], [1, 2, 5, 6, 7], [1, 2, 5, 6, 8], [1, 2, 5, 6, 9], [1, 2, 5, 6, 10], [1, 2, 5, 7, 8], [1, 2, 5, 7, 9], [1, 2, 5, 7, 10], [1, 2, 5, 8, 9], [1, 2, 6, 7, 8], [1, 2, 6, 7, 9], [1, 3, 5, 6, 7], [1, 3, 5, 6, 9], [1, 3, 5, 7, 8], [1, 3, 5, 7, 9], [1, 3, 5, 7, 10], [1, 3, 5, 8, 9], [1, 3, 5, 8, 10], [1, 3, 5, 9, 10], [1, 3, 7, 8, 9], [1, 3, 7, 9, 10], [1, 4, 5, 6, 7], [1, 4, 5, 6, 8], [1, 4, 5, 6, 9], [1, 4, 5, 6, 10], [1, 4, 5, 7, 8], [1, 4, 5, 7, 9], [1, 4, 5, 7, 10], [1, 4, 5, 8, 9], [1, 4, 5, 8, 10], [1, 4, 6, 7, 8], [1, 4, 6, 7, 10], [1, 4, 6, 8, 10], [1, 5, 6, 7, 8], [1, 5, 6, 7, 9], [1, 5, 6, 7, 10], [1, 5, 6, 8, 9], [1, 5, 6, 8, 10], [1, 5, 6, 9, 10], [1, 5, 7, 8, 9], [1, 5, 7, 8, 10], [1, 5, 7, 9, 10], [1, 5, 8, 9, 10], [1, 6, 7, 8, 9], [1, 6, 7, 8, 10], [1, 6, 7, 9, 10], [1, 6, 8, 9, 10], [1, 7, 8, 9, 10], [5, 6, 7, 8, 9], [5, 6, 7, 9, 10]
Enantiomers: [1, 2, 3, 5, 7] <-> [1, 2, 3, 6, 8]; [1, 2, 3, 5, 8] <-> [1, 2, 3, 6, 7]; [1, 2, 3, 5, 9] <-> [1, 2, 3, 6, 10]; [1, 2, 3, 5, 10] <-> [1, 2, 3, 6, 9]; [1, 2, 3, 7, 9] <-> [1, 2, 3, 8, 10]; [1, 2, 3, 7, 10] <-> [1, 2, 3, 8, 9]; [1, 2, 5, 6, 7] <-> [1, 2, 5, 6, 8]; [1, 2, 5, 6, 9] <-> [1, 2, 5, 6, 10]; [1, 2, 5, 7, 8] <-> [1, 2, 6, 7, 8]; [1, 2, 5, 7, 10] <-> [1, 2, 6, 7, 9]; [1, 4, 5, 6, 7] <-> [1, 4, 5, 6, 8]; [1, 4, 5, 6, 9] <-> [1, 4, 5, 6, 10]; [1, 4, 5, 7, 8] <-> [1, 4, 6, 7, 8]; [1, 4, 5, 7, 9] <-> [1, 4, 6, 8, 10]; [1, 4, 5, 8, 9] <-> [1, 4, 6, 7, 10]; [1, 5, 6, 7, 9] <-> [1, 5, 6, 8, 10]; [1, 5, 6, 7, 10] <-> [1, 5, 6, 8, 9]; [1, 5, 7, 8, 9] <-> [1, 6, 7, 8, 10]; [1, 5, 7, 8, 10] <-> [1, 6, 7, 8, 9]; [1, 5, 7, 9, 10] <-> [1, 6, 8, 9, 10]; [1, 5, 8, 9, 10] <-> [1, 6, 7, 9, 10]

x = 6: 60 unique configurations, 19 enantiomeric pairs

[1, 2, 3, 4, 5, 6], [1, 2, 3, 4, 5, 7], [1, 2, 3, 4, 5, 8], [1, 2, 3, 4, 5, 9], [1, 2, 3, 4, 5, 10], [1, 2, 3, 4, 7, 8], [1, 2, 3, 5, 6, 7], [1, 2, 3, 5, 6, 8], [1, 2, 3, 5, 6, 9], [1, 2, 3, 5, 6, 10], [1, 2, 3, 5, 7, 8], [1, 2, 3, 5, 7, 9], [1, 2, 3, 5, 7, 10], [1, 2, 3, 5, 8, 9], [1, 2, 3, 5, 8, 10], [1, 2, 3, 5, 9, 10], [1, 2, 3, 6, 7, 8], [1, 2, 3, 6, 7, 9], [1, 2, 3, 6, 7, 10], [1, 2, 3, 6, 8, 9], [1, 2, 3, 6, 8, 10], [1, 2, 3, 6, 9,

10], [1, 2, 3, 7, 8, 9], [1, 2, 3, 7, 8, 10], [1, 2, 3, 7, 9, 10], [1, 2, 3, 8, 9, 10], [1, 2, 5, 6, 7, 8], [1, 2, 5, 6, 7, 9], [1, 2, 5, 6, 7, 10], [1, 2, 5, 6, 8, 9], [1, 2, 5, 6, 8, 10], [1, 2, 5, 6, 9, 10], [1, 2, 5, 7, 8, 9], [1, 2, 5, 7, 8, 10], [1, 2, 6, 7, 8, 9], [1, 3, 5, 6, 7, 8], [1, 3, 5, 6, 7, 9], [1, 3, 5, 6, 7, 10], [1, 3, 5, 6, 9, 10], [1, 3, 5, 7, 8, 9], [1, 3, 5, 7, 8, 10], [1, 3, 5, 7, 9, 10], [1, 3, 5, 8, 9, 10], [1, 3, 7, 8, 9, 10], [1, 4, 5, 6, 7, 8], [1, 4, 5, 6, 7, 9], [1, 4, 5, 6, 7, 10], [1, 4, 5, 6, 8, 9], [1, 4, 5, 6, 8, 10], [1, 4, 5, 6, 9, 10], [1, 4, 5, 7, 8, 9], [1, 4, 5, 7, 8, 10], [1, 4, 6, 7, 8, 10], [1, 5, 6, 7, 8, 9], [1, 5, 6, 7, 8, 10], [1, 5, 6, 7, 9, 10], [1, 5, 6, 8, 9, 10], [1, 5, 7, 8, 9, 10], [1, 6, 7, 8, 9, 10], [5, 6, 7, 8, 9, 10]
Enantiomers: [1, 2, 3, 5, 6, 7] <-> [1, 2, 3, 5, 6, 8]; [1, 2, 3, 5, 6, 9] <-> [1, 2, 3, 5, 6, 10]; [1, 2, 3, 5, 7, 8] <-> [1, 2, 3, 6, 7, 8]; [1, 2, 3, 5, 7, 9] <-> [1, 2, 3, 6, 8, 10]; [1, 2, 3, 5, 7, 10] <-> [1, 2, 3, 6, 8, 9]; [1, 2, 3, 5, 8, 9] <-> [1, 2, 3, 6, 7, 10]; [1, 2, 3, 5, 8, 10] <-> [1, 2, 3, 6, 7, 9]; [1, 2, 3, 5, 9, 10] <-> [1, 2, 3, 6, 9, 10]; [1, 2, 3, 7, 8, 9] <-> [1, 2, 3, 7, 8, 10]; [1, 2, 3, 7, 9, 10] <-> [1, 2, 3, 8, 9, 10]; [1, 2, 5, 6, 7, 9] <-> [1, 2, 5, 6, 8, 10]; [1, 2, 5, 6, 7, 10] <-> [1, 2, 5, 6, 8, 9]; [1, 2, 5, 7, 8, 10] <-> [1, 2, 6, 7, 8, 9]; [1, 4, 5, 6, 7, 9] <-> [1, 4, 5, 6, 8, 10]; [1, 4, 5, 6, 7, 10] <-> [1, 4, 5, 6, 8, 9]; [1, 4, 5, 7, 8, 9] <-> [1, 4, 6, 7, 8, 10]; [1, 5, 6, 7, 8, 9] <-> [1, 5, 6, 7, 8, 10]; [1, 5, 6, 7, 9, 10] <-> [1, 5, 6, 8, 9, 10]; [1, 5, 7, 8, 9, 10] <-> [1, 6, 7, 8, 9, 10]

x = 7: 32 unique configurations, 9 enantiomeric pairs

[1, 2, 3, 4, 5, 6, 7], [1, 2, 3, 4, 5, 6, 9], [1, 2, 3, 4, 5, 7, 8], [1, 2, 3, 4, 5, 7, 9], [1, 2, 3, 4, 5, 7, 10], [1, 2, 3, 4, 5, 8, 9], [1, 2, 3, 5, 6, 7, 8], [1, 2, 3, 5, 6, 7, 9], [1, 2, 3, 5, 6, 7, 10], [1, 2, 3, 5, 6, 8, 9], [1, 2, 3, 5, 6, 8, 10], [1, 2, 3, 5, 6, 9, 10], [1, 2, 3, 5, 7, 8, 9], [1, 2, 3, 5, 7, 8, 10], [1, 2, 3, 5, 7, 9, 10], [1, 2, 3, 5, 8, 9, 10], [1, 2, 3, 6, 7, 8, 9], [1, 2, 3, 6, 7, 8, 10], [1, 2, 3, 6, 7, 9, 10], [1, 2, 3, 6, 8, 9, 10], [1, 2, 3, 7, 8, 9, 10], [1, 2, 5, 6, 7, 8, 9], [1, 2, 5, 6, 7, 8, 10], [1, 2, 5, 6, 7, 9, 10], [1, 3, 5, 6, 7, 8, 9], [1, 3, 5, 6, 7, 9, 10], [1, 3, 5, 7, 8, 9, 10], [1, 4, 5, 6, 7, 8, 9], [1, 4, 5, 6, 7, 8, 10], [1, 4, 5, 6, 7, 9, 10], [1, 4, 5, 6, 8, 9, 10], [1, 5, 6, 7, 8, 9, 10]
Enantiomers: [1, 2, 3, 5, 6, 7, 9] <-> [1, 2, 3, 5, 6, 8, 10]; [1, 2, 3, 5, 6, 7, 10] <-> [1, 2, 3, 5, 6, 8, 9]; [1, 2, 3, 5, 7, 8, 9] <-> [1, 2, 3, 6, 7, 8, 10]; [1, 2, 3, 5, 7, 8, 10] <-> [1, 2, 3, 6, 7, 8, 9]; [1, 2, 3, 5, 7, 9, 10] <-> [1, 2, 3, 6, 8, 9, 10]; [1, 2, 3, 5, 8, 9, 10] <-> [1, 2, 3, 6, 7, 9, 10]; [1, 2, 5, 6, 7, 8, 9] <-> [1, 2, 5, 6, 7, 8, 10]; [1, 4, 5, 6, 7, 8, 9] <-> [1, 4, 5, 6, 7, 8, 10]; [1, 4, 5, 6, 7, 9, 10] <-> [1, 4, 5, 6, 8, 9, 10]

x = 8: 15 unique configurations, 3 enantiomeric pairs

[1, 2, 3, 4, 5, 6, 7, 8], [1, 2, 3, 4, 5, 6, 7, 9], [1, 2, 3, 4, 5, 6, 7, 10], [1, 2, 3, 4, 5, 6, 9, 10], [1, 2, 3, 4, 5, 7, 8, 9], [1, 2, 3, 4, 5, 7, 8, 10], [1, 2, 3, 5, 6, 7, 8, 9], [1, 2, 3, 5, 6, 7, 8, 10], [1, 2, 3, 5, 6, 7, 9, 10], [1, 2, 3, 5, 6, 8, 9, 10], [1, 2, 3, 5, 7, 8, 9, 10], [1, 2, 3, 6, 7, 8, 9, 10], [1, 2, 5, 6, 7, 8, 9, 10], [1, 3, 5, 6, 7, 8, 9, 10], [1, 4, 5, 6, 7, 8, 9, 10]
Enantiomers: [1, 2, 3, 5, 6, 7, 8, 9] <-> [1, 2, 3, 5, 6, 7, 8, 10]; [1, 2, 3, 5, 6, 7, 9, 10] <-> [1, 2, 3, 5, 6, 8, 9, 10]; [1, 2, 3, 5, 7, 8, 9, 10] <-> [1, 2, 3, 6, 7, 8, 9, 10]

x = 9: 3 unique configurations, 0 enantiomeric pairs

[1, 2, 3, 4, 5, 6, 7, 8, 9], [1, 2, 3, 4, 5, 6, 7, 9, 10], [1, 2, 3, 5, 6, 7, 8, 9, 10]

x = 10: 1 unique configurations, 0 enantiomeric pairs

[1, 2, 3, 4, 5, 6, 7, 8, 9, 10]

2 DFT calculations

Pos.	HOMO	LUMO	$\Delta_{LUMO-HOMO}$ (eV)	E (kJ/mol)	Dipole	Δ (kJ/mol)	Type
1	-7.18	-3.35	3.83	-38124.66	1.22	0.00	A
5	-7.16	-3.47	3.68	-38109.27	1.60	15.39	B
7	-7.17	-3.35	3.83	-38103.81	0.41	20.85	C

Table 1: Summary of HOMO, LUMO, HOMO-LUMO Gap, Bonding Energy, Dipole Moment, Relative Bonding Energy, and Atom Type for Various Calculations

Pos.	HOMO	LUMO	$\Delta_{LUMO-HOMO}$ (eV)	E (kJ/mol)	Δ (kJ/mol)	Dipole	Type
1_4	-7.67	-3.74	3.93	-37826.42	0.00	0.000	AA
1_2	-7.65	-3.73	3.92	-37825.83	0.59	1.323	AA
1_3	-7.56	-3.86	3.70	-37815.84	10.58	2.452	AA
1_10	-7.62	-3.81	3.81	-37813.35	13.07	1.070	AB
1_9	-7.62	-3.81	3.81	-37813.35	13.07	1.070	AB
1_5	-7.56	-3.88	3.68	-37805.85	20.57	2.810	AB
1_6	-7.56	-3.88	3.68	-37805.85	20.57	2.810	AB
1_7	-7.61	-3.77	3.84	-37802.65	23.77	1.560	AC
1_8	-7.61	-3.77	3.84	-37802.65	23.77	1.560	AC
5_10	-7.59	-3.80	3.79	-37799.03	27.38	0.000	BB
5_9	-7.57	-3.81	3.76	-37797.07	29.35	1.081	BB
5_7	-7.59	-3.89	3.70	-37791.43	34.99	2.022	BC
5_6	-7.53	-3.93	3.60	-37788.23	38.19	3.192	BB
5_8	-7.57	-3.81	3.76	-37784.74	41.68	1.938	BC
7_8	-7.59	-3.72	3.87	-37778.46	47.96	0.000	CC

Table 2: Summary of HOMO, LUMO, HOMO-LUMO Gap, Bonding Energy, Relative Bonding Energy, Dipole Moment, and AtomTypeOccupation for Various Calculations

Pos.	HOMO	LUMO	$\Delta_{LUMO-HOMO}$ (eV)	E (kJ/mol)	Δ (kJ/mol)	Dipole	Type
1_2_3	-7.02	-4.83	2.19	-30920.68	0.00	1.542	AAA
1_3_9	-6.95	-4.81	2.14	-30916.05	4.63	0.815	AAB
1_4_6	-6.99	-4.88	2.11	-30915.28	5.39	1.857	AAB
1_4_5	-6.99	-4.88	2.11	-30915.28	5.40	1.856	AAB
1_2_6	-7.01	-4.86	2.15	-30914.49	6.19	2.225	AAB
1_2_5	-7.01	-4.86	2.15	-30914.48	6.19	2.225	AAB
1_6_9	-6.94	-4.83	2.10	-30908.12	12.55	1.098	ABB
1_5_10	-6.94	-4.83	2.10	-30908.12	12.56	1.098	ABB
1_5_9	-6.90	-4.83	2.07	-30906.83	13.85	1.656	ABB
1_6_10	-6.90	-4.84	2.07	-30906.82	13.85	1.631	ABB
1_9_10	-6.92	-4.89	2.02	-30904.68	15.99	2.36	ABB
1_4_7	-7.07	-4.74	2.32	-30902.99	17.69	0.532	AAC
1_4_8	-7.07	-4.74	2.32	-30902.99	17.69	0.532	AAC
1_2_7	-7.09	-4.75	2.35	-30902.29	18.39	1.599	AAC
1_3_5	-6.96	-4.99	1.97	-30897.63	23.05	4.044	AAB
1_7_9	-7.03	-4.86	2.17	-30896.75	23.92	1.494	ACB
1_8_10	-7.03	-4.86	2.17	-30896.75	23.93	1.494	ACB
5_6_9	-6.86	-4.85	2.01	-30893.86	26.81	1.581	BBB
1_3_7	-7.02	-4.90	2.12	-30893.45	27.23	2.789	AAC
1_7_10	-6.99	-4.79	2.21	-30892.59	28.09	1.106	ACB
1_8_9	-6.99	-4.79	2.21	-30892.59	28.09	1.106	ACB
1_5_6	-6.97	-4.92	2.05	-30889.54	31.13	4.289	ABB
5_7_9	-6.97	-4.86	2.11	-30887.37	33.30	1.624	BCB
1_6_8	-7.02	-4.93	2.09	-30886.45	34.23	3.299	ABC
1_5_7	-7.02	-4.93	2.09	-30886.45	34.23	3.299	ABC
5_7_10	-6.96	-4.84	2.12	-30884.83	35.85	0.501	BCB
1_5_8	-7.00	-4.87	2.13	-30882.58	38.10	3.225	ABC
1_6_7	-7.00	-4.87	2.13	-30882.57	38.11	3.225	ABC
5_8_9	-6.91	-4.78	2.13	-30878.94	41.73	0.723	BCB
1_7_8	-7.07	-4.75	2.32	-30873.95	46.72	1.655	ACC
5_6_7	-6.99	-4.95	2.04	-30870.95	49.72	3.67	BBC
5_7_8	-7.03	-4.86	2.17	-30862.68	57.99	2.251	BCC

Table 3: Summary of HOMO, LUMO, HOMO-LUMO Gap, Bonding Energy, Relative Bonding Energy, and Atom-Type for Various Calculations

Table 4: Geometric parameters for $[Mo_xV_{10-x}O_{28}]^{n-}$

Configuration	μ_n	d(V-O) Å	d(Mo-O) Å	Number of O atoms
0	1	1.6331-1.6370	None-None	8
0	2	1.7206-2.0332	None-None	14
0	3	1.9357-2.0230	None-None	4
0	6	2.0957-2.3793	None-None	2
1	1	1.6241-1.6315	1.7372-1.7372	8
1	2	1.7084-2.0483	1.8654-1.8654	14
1	3	1.9171-2.0556	2.0635-2.0635	4
1	6	2.0689-2.4230	2.3247-2.3247	2
5	1	1.6219-1.6301	1.7351-1.7351	8
5	2	1.6913-2.0926	1.8846-2.1023	14
5	3	1.9309-2.0697	None-None	4
5	6	2.0025-2.4781	2.3359-2.3359	2
7	1	1.6224-1.6291	None-None	8
7	2	1.7160-2.1352	1.7955-1.7955	14
7	3	1.9312-2.0938	1.9613-1.9613	4
7	6	2.1105-2.4572	2.1543-2.1543	2
1_2	1	1.6167-1.6180	1.7307-1.7307	8
1_2	2	1.7151-2.0164	1.8541-1.8541	14
1_2	3	1.8986-2.0263	2.0913-2.0913	4
1_2	6	2.0886-2.4149	2.3298-2.3298	2
1_3	1	1.6141-1.6251	1.7251-1.7251	8
1_3	2	1.6969-2.0639	1.8808-1.8808	14
1_3	3	1.9581-2.0818	2.0426-2.0426	4
1_3	6	2.0483-2.4659	2.3724-2.3724	2
1_4	1	1.6167-1.6197	1.7296-1.7296	8
1_4	2	1.7136-2.0206	1.8573-1.8573	14
1_4	3	1.9568-2.0299	2.0844-2.0844	4
1_4	6	2.0917-2.4124	2.3345-2.3345	2
1_5	1	1.6142-1.6242	1.7250-1.7285	8
1_5	2	1.6850-2.0931	1.8565-2.0586	14
1_5	3	1.9169-2.1069	2.0517-2.0720	4
1_5	6	2.0001-2.5128	2.3261-2.3449	2
1_6	1	1.6142-1.6242	1.7250-1.7285	8
1_6	2	1.6850-2.0930	1.8565-2.0585	14
1_6	3	1.9169-2.1069	2.0517-2.0720	4
1_6	6	2.0002-2.5128	2.3261-2.3450	2
1_7	1	1.6138-1.6243	1.7287-1.7287	8
1_7	2	1.7050-2.1532	1.7855-1.8728	14
1_7	3	1.9082-2.1228	1.9427-2.1260	4
1_7	6	2.0979-2.5003	2.1239-2.3242	2
1_8	1	1.6138-1.6243	1.7287-1.7287	8

Continued on next page

Table 4 – *Continued from previous page*

Configuration	μ_n	d(V-O) Å	d(Mo-O) Å	Number of O atoms
1_8	2	1.7050-2.1532	1.7855-1.8728	14
1_8	3	1.9083-2.1228	1.9426-2.1261	4
1_8	6	2.0979-2.5002	2.1239-2.3242	2
1_9	1	1.6148-1.6214	1.7252-1.7304	8
1_9	2	1.7036-2.0365	1.8577-2.0835	14
1_9	3	1.8998-2.0757	2.0521-2.1006	4
1_9	6	2.0611-2.4535	2.3284-2.3947	2
1_10	1	1.6148-1.6214	1.7252-1.7304	8
1_10	2	1.7036-2.0365	1.8577-2.0835	14
1_10	3	1.8998-2.0756	2.0521-2.1006	4
1_10	6	2.0611-2.4535	2.3284-2.3948	2
5_6	1	1.6176-1.6238	1.7237-1.7237	8
5_6	2	1.6863-2.0711	1.9234-2.0155	14
5_6	3	1.9297-2.0669	None-None	4
5_6	6	2.0194-2.4930	2.3707-2.3707	2
5_7	1	1.6123-1.6229	1.7268-1.7268	8
5_7	2	1.6905-2.1296	1.7885-2.1787	14
5_7	3	1.9284-2.1084	1.9576-1.9576	4
5_7	6	2.0406-2.5059	2.1412-2.3588	2
5_8	1	1.6148-1.6230	1.7226-1.7226	8
5_8	2	1.7064-2.2030	1.7748-2.0605	14
5_8	3	1.9272-2.1320	1.9637-1.9637	4
5_8	6	2.1051-2.5436	2.0824-2.4140	2
5_9	1	1.6156-1.6210	1.7241-1.7241	8
5_9	2	1.7161-1.9871	1.8898-2.0765	14
5_9	3	1.9266-2.0303	None-None	4
5_9	6	2.0860-2.4232	2.4304-2.4304	2
5_10	1	1.6141-1.6211	1.7256-1.7256	8
5_10	2	1.7214-1.9706	1.8957-2.0991	14
5_10	3	1.9321-2.0838	None-None	4
5_10	6	2.0658-2.4712	2.3969-2.3969	2
7_8	1	1.6157-1.6218	None-None	8
7_8	2	1.8128-2.0985	1.7909-1.7909	14
7_8	3	2.0852-2.0852	1.9590-1.9590	4
7_8	6	2.2823-2.4825	2.1673-2.1673	2
1_2_3	1	1.6069-1.6140	1.7180-1.7242	8
1_2_3	2	1.7038-2.0323	1.8477-1.8714	14
1_2_3	3	1.9349-2.0578	2.0640-2.1118	4
1_2_3	6	2.0732-2.4503	2.3386-2.3872	2
1_2_5	1	1.6069-1.6127	1.7163-1.7240	8
1_2_5	2	1.6943-2.0455	1.8510-2.0514	14
1_2_5	3	1.8861-2.0694	2.0670-2.1142	4

Continued on next page

Table 4 – *Continued from previous page*

Configuration	μ_n	d(V-O) Å	d(Mo-O) Å	Number of O atoms
1_2_5	6	2.0408-2.4934	2.3350-2.3920	2
1_2_6	1	1.6069-1.6127	1.7163-1.7240	8
1_2_6	2	1.6943-2.0455	1.8510-2.0514	14
1_2_6	3	1.8861-2.0694	2.0669-2.1142	4
1_2_6	6	2.0408-2.4934	2.3350-2.3920	2
1_2_7	1	1.6073-1.6116	1.7227-1.7227	8
1_2_7	2	1.7107-2.1080	1.7904-1.8586	14
1_2_7	3	1.8917-2.0989	1.9227-2.1426	4
1_2_7	6	2.1063-2.4949	2.1480-2.3316	2
1_3_5	1	1.6043-1.6189	1.7156-1.7163	8
1_3_5	2	1.6746-2.1096	1.8744-2.0456	14
1_3_5	3	1.9511-2.1308	2.0047-2.0470	4
1_3_5	6	1.9836-2.5542	2.3505-2.3823	2
1_3_7	1	1.6040-1.6185	1.7173-1.7173	8
1_3_7	2	1.6925-2.1802	1.7760-1.8822	14
1_3_7	3	1.9490-2.1423	1.9800-2.1085	4
1_3_7	6	2.0817-2.5431	2.1014-2.3679	2
1_3_9	1	1.6068-1.6158	1.7175-1.7192	8
1_3_9	2	1.7119-2.0062	1.8720-2.0884	14
1_3_9	3	1.9533-2.0998	2.0384-2.0773	4
1_3_9	6	2.0822-2.4725	2.3774-2.4428	2
1_4_5	1	1.6068-1.6140	1.7164-1.7231	8
1_4_5	2	1.6921-2.0533	1.8493-2.0608	14
1_4_5	3	1.9387-2.0599	2.0592-2.1208	4
1_4_5	6	2.0354-2.4934	2.3330-2.3912	2
1_4_6	1	1.6067-1.6140	1.7164-1.7231	8
1_4_6	2	1.6919-2.0539	1.8492-2.0612	14
1_4_6	3	1.9388-2.0599	2.0593-2.1212	4
1_4_6	6	2.0350-2.4936	2.3333-2.3906	2
1_4_7	1	1.6076-1.6131	1.7219-1.7219	8
1_4_7	2	1.7094-2.1145	1.7891-1.8648	14
1_4_7	3	1.9500-2.0908	1.9783-2.1488	4
1_4_7	6	2.1098-2.4953	2.1527-2.3354	2
1_4_8	1	1.6076-1.6131	1.7219-1.7219	8
1_4_8	2	1.7094-2.1146	1.7891-1.8648	14
1_4_8	3	1.9500-2.0908	1.9783-2.1488	4
1_4_8	6	2.1097-2.4953	2.1528-2.3354	2
1_5_6	1	1.6062-1.6181	1.7154-1.7197	8
1_5_6	2	1.6746-2.0956	1.8741-2.0302	14
1_5_6	3	1.9208-2.1055	2.0513-2.0513	4
1_5_6	6	1.9966-2.5456	2.3378-2.3608	2
1_5_7	1	1.6045-1.6181	1.7176-1.7205	8

Continued on next page

Table 4 – *Continued from previous page*

Configuration	μ_n	d(V-O) Å	d(Mo-O) Å	Number of O atoms
1_5_7	2	1.6804-2.1589	1.7769-2.1476	14
1_5_7	3	1.9070-2.1141	1.9537-2.1503	4
1_5_7	6	2.0258-2.5555	2.1148-2.3582	2
1_5_8	1	1.6062-1.6181	1.7138-1.7202	8
1_5_8	2	1.6907-2.2149	1.7675-2.0484	14
1_5_8	3	1.8894-2.1543	1.9651-2.1275	4
1_5_8	6	2.0698-2.5748	2.0747-2.4210	2
1_5_9	1	1.6075-1.6158	1.7156-1.7220	8
1_5_9	2	1.7045-1.9987	1.8590-2.0919	14
1_5_9	3	1.9082-2.0734	2.0612-2.0620	4
1_5_9	6	2.0568-2.4766	2.3408-2.4647	2
1_5_10	1	1.6068-1.6152	1.7169-1.7223	8
1_5_10	2	1.7103-1.9889	1.8487-2.1053	14
1_5_10	3	1.9133-2.1237	2.0219-2.1118	4
1_5_10	6	2.0403-2.5157	2.3321-2.4472	2
1_6_7	1	1.6062-1.6181	1.7138-1.7202	8
1_6_7	2	1.6907-2.2151	1.7674-2.0487	14
1_6_7	3	1.8892-2.1541	1.9648-2.1278	4
1_6_7	6	2.0699-2.5749	2.0746-2.4210	2
1_6_8	1	1.6045-1.6181	1.7176-1.7205	8
1_6_8	2	1.6805-2.1591	1.7769-2.1475	14
1_6_8	3	1.9070-2.1141	1.9537-2.1503	4
1_6_8	6	2.0259-2.5554	2.1147-2.3583	2
1_6_9	1	1.6068-1.6152	1.7169-1.7223	8
1_6_9	2	1.7103-1.9889	1.8486-2.1053	14
1_6_9	3	1.9133-2.1237	2.0219-2.1118	4
1_6_9	6	2.0403-2.5157	2.3321-2.4472	2
1_6_10	1	1.6074-1.6157	1.7157-1.7219	8
1_6_10	2	1.7041-2.0000	1.8588-2.0905	14
1_6_10	3	1.9068-2.0723	2.0613-2.0669	4
1_6_10	6	2.0550-2.4805	2.3376-2.4669	2
1_7_8	1	1.6071-1.6175	1.7209-1.7209	8
1_7_8	2	1.8014-2.1191	1.7812-1.8568	14
1_7_8	3	2.0693-2.1210	1.9361-2.1077	4
1_7_8	6	2.2919-2.5292	2.1475-2.3291	2
1_7_9	1	1.6058-1.6147	1.7181-1.7228	8
1_7_9	2	1.6996-2.0995	1.7901-2.1742	14
1_7_9	3	1.9069-2.1454	1.9361-2.1213	4
1_7_9	6	2.0918-2.5125	2.1505-2.4172	2
1_7_10	1	1.6075-1.6143	1.7143-1.7229	8
1_7_10	2	1.7053-2.1195	1.7842-2.0549	14
1_7_10	3	1.8969-2.0950	1.9467-2.1645	4

Continued on next page

Table 4 – *Continued from previous page*

Configuration	μ_n	d(V-O) Å	d(Mo-O) Å	Number of O atoms
1_7_10	6	2.0907-2.5125	2.1446-2.4853	2
1_8_9	1	1.6075-1.6143	1.7143-1.7229	8
1_8_9	2	1.7053-2.1195	1.7842-2.0549	14
1_8_9	3	1.8969-2.0950	1.9467-2.1644	4
1_8_9	6	2.0906-2.5125	2.1446-2.4852	2
1_8_10	1	1.6058-1.6147	1.7181-1.7228	8
1_8_10	2	1.6996-2.0995	1.7901-2.1741	14
1_8_10	3	1.9069-2.1454	1.9360-2.1214	4
1_8_10	6	2.0918-2.5124	2.1505-2.4172	2
1_9_10	1	1.6097-1.6131	1.7158-1.7236	8
1_9_10	2	1.6916-2.0421	1.8569-2.0218	14
1_9_10	3	1.9091-2.0410	2.0940-2.0940	4
1_9_10	6	2.0401-2.4833	2.3382-2.4054	2
5_6_7	1	1.6100-1.6173	1.7125-1.7162	8
5_6_7	2	1.6783-2.1748	1.7704-2.1040	14
5_6_7	3	1.9230-2.1254	1.9585-1.9585	4
5_6_7	6	2.0304-2.5609	2.0979-2.4464	2
5_6_9	1	1.6079-1.6148	1.7152-1.7168	8
5_6_9	2	1.6993-2.0056	1.8931-2.0924	14
5_6_9	3	1.9270-2.0861	None-None	4
5_6_9	6	2.0554-2.4960	2.4211-2.4718	2
5_7_8	1	1.6050-1.6165	1.7152-1.7152	8
5_7_8	2	1.8019-2.1463	1.7726-2.1377	14
5_7_8	3	2.0110-2.1340	1.9552-1.9603	4
5_7_8	6	2.2890-2.5703	2.1109-2.4380	2
5_7_9	1	1.6065-1.6141	1.7173-1.7173	8
5_7_9	2	1.7104-1.9709	1.8048-2.1584	14
5_7_9	3	1.9288-2.1025	1.9533-1.9533	4
5_7_9	6	2.1315-2.5065	2.1504-2.4473	2
5_7_10	1	1.6056-1.6144	1.7152-1.7190	8
5_7_10	2	1.7172-2.0445	1.7948-2.1922	14
5_7_10	3	1.9260-2.1520	1.9592-1.9592	4
5_7_10	6	2.0748-2.5398	2.1307-2.4734	2
5_8_9	1	1.6090-1.6137	1.7141-1.7141	8
5_8_9	2	1.7281-2.0720	1.7901-2.0572	14
5_8_9	3	1.9256-2.0560	1.9660-1.9660	4
5_8_9	6	2.0943-2.4547	2.1619-2.5042	2

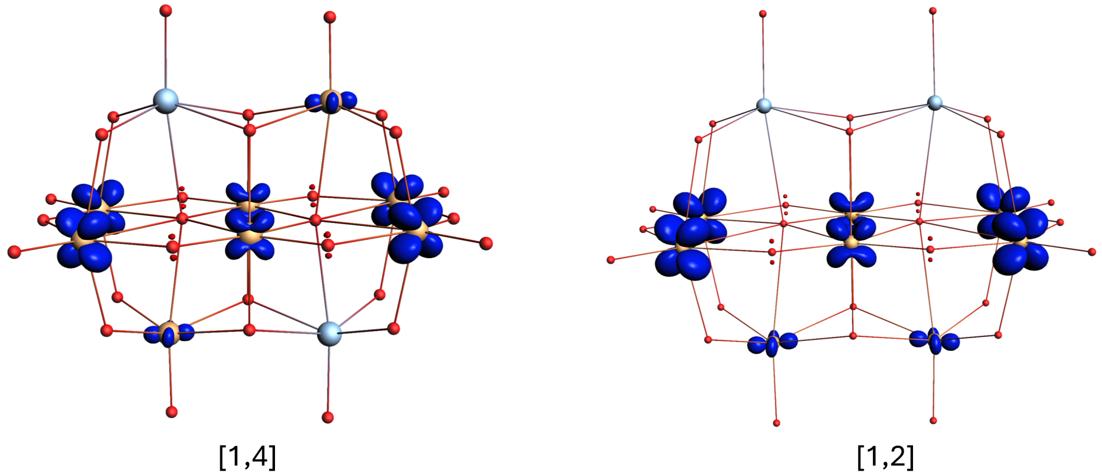

Figure 1: Spin density isosurface of [1,4] and [1,2] ($[Mo_2V_8O_{28}]^{6-}$)

3 Optimised Geometries

All geometries are optimised at BP86/ZORA-scalar/TZP/COSMO-water level (closed shell) unless specified otherwise.
$[V_{10}O_{28}]^{6-}$ Symmol = D(2H)

V	1.63492962	-0.00000000	0.00000000
O	2.65345220	-0.00000000	1.38670168
O	2.65345220	-0.00000000	-1.38670168
O	1.24088431	-1.89511754	0.00000000
O	1.24088431	1.89511754	0.00000000
O	0.00000000	0.00000000	1.31114022
O	-0.00000000	0.00000000	-1.31114022
V	1.56458366	-0.00000000	3.10371640
V	1.56458366	-0.00000000	-3.10371640
V	-0.00000000	-2.26452299	1.55449828
V	-0.00000000	-2.26452299	-1.55449828
V	0.00000000	2.26452299	-1.55449828
V	0.00000000	2.26452299	1.55449828
V	-1.63492962	0.00000000	0.00000000
V	-1.56458366	0.00000000	3.10371640
V	-1.56458366	0.00000000	-3.10371640
O	1.34030066	-1.82533645	2.71719646
O	1.34030066	1.82533645	2.71719646
O	2.74794243	-0.00000000	4.22913713
O	-0.00000000	0.00000000	4.08053778
O	1.34030066	-1.82533645	-2.71719646
O	1.34030066	1.82533645	-2.71719646
O	0.00000000	0.00000000	-4.08053778
O	2.74794243	-0.00000000	-4.22913713
O	-1.24088431	-1.89511754	0.00000000
O	-0.00000000	-3.90144766	1.56901329
O	-1.34030066	-1.82533645	2.71719646
O	-0.00000000	-3.90144766	-1.56901329
O	-1.34030066	-1.82533645	-2.71719646
O	-1.24088431	1.89511754	0.00000000
O	-1.34030066	1.82533645	-2.71719646
O	0.00000000	3.90144766	-1.56901329
O	-1.34030066	1.82533645	2.71719646
O	0.00000000	3.90144766	1.56901329
O	-2.65345220	0.00000000	-1.38670168
O	-2.65345220	0.00000000	1.38670168
O	-2.74794243	0.00000000	4.22913713
O	-2.74794243	0.00000000	-4.22913713

[1] - $[MoV_9O_{28}]^{5-}$ Symmol = C(S)

Mo	1.58127267	2.34546763	0.00000000
V	-1.60219681	2.26886841	0.00000000
V	1.52959572	-2.29321330	0.00000000
V	-1.56802675	-2.27171450	0.00000000
V	3.10491781	-0.07077979	1.57367214
V	3.10491781	-0.07077979	-1.57367214
V	-0.00762132	-0.01538241	1.63432818
V	-0.00762132	-0.01538241	-1.63432818
V	-3.12164708	-0.00932281	1.56981589
V	-3.12164708	-0.00932281	-1.56981589
O	1.40196898	0.00841899	-2.63095483
O	-1.38132958	0.00298194	-2.64989893
O	0.01785033	-1.89143026	-1.24042024
O	-0.00640657	1.92560854	-1.24948721
O	1.33461712	0.03388796	0.00000000
O	-1.27602270	0.00885526	0.00000000
O	2.72883201	-1.85402573	-1.33295400
O	2.74484021	1.85771605	-1.37395430
O	4.23168288	-0.05235133	-2.74314599
O	4.06121076	0.01359376	0.00000000
O	-2.70946665	-1.82841226	-1.34133543
O	-2.73452924	1.81642095	-1.34369392
O	-4.08146194	-0.01414806	0.00000000
O	-4.24050898	-0.01716805	-2.74874144
O	0.01785033	-1.89143026	1.24042024
O	1.54516692	-3.91766901	0.00000000
O	2.72883201	-1.85402573	1.33295400
O	-1.56381369	-3.90142916	0.00000000
O	-2.70946665	-1.82841226	1.34133543
O	-0.00640657	1.92560854	1.24948721
O	-2.73452924	1.81642095	1.34369392
O	-1.60636630	3.90034805	0.00000000
O	2.74484021	1.85771605	1.37395430
O	1.58885946	4.08260528	0.00000000
O	-1.38132958	0.00298194	2.64989893
O	1.40196898	0.00841899	2.63095483
O	4.23168288	-0.05235133	2.74314599
O	-4.24050898	-0.01716805	2.74874144

[5] - $[MoV_9O_{28}]^{5-}$ Symmol = C(S)

V	0.04928744	1.52207460	-2.27661603
V	-0.01631427	-1.56915704	-2.26711219
V	0.04928744	1.52207460	2.27661603
V	-0.01631427	-1.56915704	2.26711219
Mo	-1.58108974	3.14440336	0.00000000
V	1.63291193	3.07070087	0.00000000
V	-1.64398373	0.00736911	0.00000000
V	1.63561365	-0.05331110	0.00000000
V	-1.58241585	-3.13572635	0.00000000
V	1.55758507	-3.12735655	0.00000000
O	2.61523278	1.40118019	0.00000000
O	2.69554204	-1.37125822	0.00000000
O	1.25186889	0.02367371	1.89540338
O	1.25186889	0.02367371	-1.89540338
O	-0.06527540	1.36717954	0.00000000
O	0.03818963	-1.26093536	0.00000000
O	1.32058400	2.74073195	1.82522992
O	1.32058400	2.74073195	-1.82522992
O	2.80389464	4.19287936	0.00000000
O	0.02702464	4.12701900	0.00000000
O	1.34157514	-2.70425331	1.81292155
O	1.34157514	-2.70425331	-1.81292155
O	-0.00264029	-4.08804478	0.00000000
O	2.74267714	-4.24149965	0.00000000
O	-1.23475280	-0.00381745	1.88695427
O	0.02423603	1.54924411	3.90339433
O	-1.36235571	2.71402361	1.86044467
O	-0.00572088	-1.57414145	3.89713707
O	-1.33508997	-2.73155273	1.81839948
O	-1.23475280	-0.00381745	-1.88695427
O	-1.33508997	-2.73155273	-1.81839948
O	-0.00572088	-1.57414145	-3.89713707
O	-1.36235571	2.71402361	-1.86044467
O	0.02423603	1.54924411	-3.90339433
O	-2.61967544	-1.40403820	0.00000000
O	-2.71241507	1.37241880	0.00000000
O	-2.85300093	4.32454742	0.00000000
O	-2.75481082	-4.25917947	0.00000000

[7] - $[MoV_9O_{28}]^{5-}$ Symmol = C(2V)

V	1.59283149	2.26159797	-0.01348472
V	-1.59283149	2.26159797	-0.01348472
V	1.59283149	-2.26159797	-0.01348472
V	-1.59283149	-2.26159797	-0.01348472
V	3.21487266	0.00000000	1.51299015
V	3.12116024	0.00000000	-1.61670016
Mo	0.00000000	-0.00000000	1.75461295
V	0.00000000	-0.00000000	-1.60797552
V	-3.21487266	-0.00000000	1.51299015
V	-3.12116024	-0.00000000	-1.61670016
O	1.38247893	0.00000000	-2.62454073
O	-1.38247893	-0.00000000	-2.62454073
O	0.00000000	-1.88581420	-1.19165473
O	-0.00000000	1.88581420	-1.19165473
O	1.31150455	0.00000000	0.04550428
O	-1.31150455	-0.00000000	0.04550428
O	2.72875255	-1.82445061	-1.35351728
O	2.72875255	1.82445061	-1.35351728
O	4.21576001	0.00000000	-2.81426987
O	4.12732511	0.00000000	-0.06433569
O	-2.72875255	-1.82445061	-1.35351728
O	-2.72875255	1.82445061	-1.35351728
O	-4.12732511	-0.00000000	-0.06433569
O	-4.21576001	-0.00000000	-2.81426987
O	0.00000000	-1.90755839	1.29855362
O	1.58882917	-3.89041064	0.01643854
O	2.75451222	-1.80396367	1.32513318
O	-1.58882917	-3.89041064	0.01643854
O	-2.75451222	-1.80396367	1.32513318
O	-0.00000000	1.90755839	1.29855362
O	-2.75451222	1.80396367	1.32513318
O	-1.58882917	3.89041064	0.01643854
O	2.75451222	1.80396367	1.32513318
O	1.58882917	3.89041064	0.01643854
O	-1.48472274	-0.00000000	2.76422719
O	1.48472274	0.00000000	2.76422719
O	4.35991389	0.00000000	2.66776778
O	-4.35991389	-0.00000000	2.66776778

[1,2] - $[Mo_2V_8O_{28}]^{4-}$ Symmol = C(2V)

Mo	-1.62683987	-0.00000000	2.34890308
Mo	1.62683987	0.00000000	2.34890308
V	-1.54669676	-0.00000000	-2.30020977
V	1.54669676	0.00000000	-2.30020977
V	-3.11978243	1.58041482	-0.07886444
V	-3.11978243	-1.58041482	-0.07886444
V	-0.00000000	1.63481875	-0.03151597
V	0.00000000	-1.63481875	-0.03151597
V	3.11978243	1.58041482	-0.07886444
V	3.11978243	-1.58041482	-0.07886444
O	-1.39851657	-2.62660919	0.01319452
O	1.39851657	-2.62660919	0.01319452
O	0.00000000	-1.23814027	-1.88819566
O	0.00000000	-1.25482545	1.95880437
O	-1.29782699	-0.00000000	0.04244892
O	1.29782699	0.00000000	0.04244892
O	-2.72054178	-1.33468945	-1.85732299
O	-2.76086843	-1.37900116	1.84892394
O	-4.24341924	-2.74281727	-0.07104473
O	-4.05630256	-0.00000000	-0.00146292
O	2.72054178	-1.33468945	-1.85732299
O	2.76086843	-1.37900116	1.84892394
O	4.05630256	0.00000000	-0.00146292
O	4.24341924	-2.74281727	-0.07104473
O	-0.00000000	1.23814027	-1.88819566
O	-1.54330157	-0.00000000	-3.91819066
O	-2.72054178	1.33468945	-1.85732299
O	1.54330157	0.00000000	-3.91819066
O	2.72054178	1.33468945	-1.85732299
O	-0.00000000	1.25482545	1.95880437
O	2.76086843	1.37900116	1.84892394
O	1.63072420	0.00000000	4.07964599
O	-2.76086843	1.37900116	1.84892394
O	-1.63072420	-0.00000000	4.07964599
O	1.39851657	2.62660919	0.01319452
O	-1.39851657	2.62660919	0.01319452
O	-4.24341924	2.74281727	-0.07104473
O	4.24341924	2.74281727	-0.07104473

[1,3] - $[Mo_2V_8O_{28}]^{4-}$ Symmol = C(2V)

Mo	-0.00000000	2.36286100	1.56100060
V	-0.00000000	2.27297852	-1.61612142
Mo	0.00000000	-2.36286100	1.56100060
V	0.00000000	-2.27297852	-1.61612142
V	-1.58822911	-0.00000000	3.11326241
V	1.58822911	0.00000000	3.11326241
V	-1.63752107	-0.00000000	-0.01470402
V	1.63752107	0.00000000	-0.01470402
V	-1.57421668	-0.00000000	-3.14312952
V	1.57421668	0.00000000	-3.14312952
O	2.61612567	0.00000000	1.41383029
O	2.64603997	0.00000000	-1.37941594
O	1.25193594	-1.91959656	0.00915882
O	1.25193594	1.91959656	0.00915882
O	0.00000000	-0.00000000	1.34868628
O	0.00000000	-0.00000000	-1.24509167
O	1.36510877	-1.86931773	2.75691240
O	1.36510877	1.86931773	2.75691240
O	2.74291271	0.00000000	4.24115806
O	0.00000000	0.00000000	4.04935342
O	1.34416367	-1.81936897	-2.73068990
O	1.34416367	1.81936897	-2.73068990
O	0.00000000	0.00000000	-4.08799598
O	2.74807087	0.00000000	-4.25736388
O	-1.25193594	-1.91959656	0.00915882
O	0.00000000	-4.08791255	1.57700772
O	-1.36510877	-1.86931773	2.75691240
O	0.00000000	-3.89796459	-1.59876299
O	-1.34416367	-1.81936897	-2.73068990
O	-1.25193594	1.91959656	0.00915882
O	-1.34416367	1.81936897	-2.73068990
O	-0.00000000	3.89796459	-1.59876299
O	-1.36510877	1.86931773	2.75691240
O	-0.00000000	4.08791255	1.57700772
O	-2.64603997	-0.00000000	-1.37941594
O	-2.61612567	-0.00000000	1.41383029
O	-2.74291271	-0.00000000	4.24115806
O	-2.74807087	-0.00000000	-4.25736388

[1,4] - $[Mo_2V_8O_{28}]^{4-}$ Symmol = C(2H)

Mo	1.58837260	2.34464084	0.00000000
V	-1.58235605	2.29684523	0.00000000
V	1.58235605	-2.29684523	0.00000000
Mo	-1.58837260	-2.34464084	0.00000000
V	3.12302416	-0.06010580	1.57972026
V	3.12302416	-0.06010580	-1.57972026
V	0.00000000	-0.00000000	1.63684401
V	-0.00000000	0.00000000	-1.63684401
V	-3.12302416	0.06010580	1.57972026
V	-3.12302416	0.06010580	-1.57972026
O	1.39729073	0.00357424	-2.62887778
O	-1.39729073	-0.00357424	-2.62887778
O	0.02624625	-1.91740084	-1.24707126
O	-0.02624625	1.91740084	-1.24707126
O	1.30198353	0.02782441	0.00000000
O	-1.30198353	-0.02782441	0.00000000
O	2.74740560	-1.84632404	-1.33704775
O	2.73541962	1.85709827	-1.37705490
O	4.24544798	-0.03767488	-2.74310516
O	4.06098942	0.02495535	0.00000000
O	-2.73541962	-1.85709827	-1.37705490
O	-2.74740560	1.84632404	-1.33704775
O	-4.06098942	-0.02495535	0.00000000
O	-4.24544798	0.03767488	-2.74310516
O	0.02624625	-1.91740084	1.24707126
O	1.58544249	-3.91653292	0.00000000
O	2.74740560	-1.84632404	1.33704775
O	-1.57793476	-4.07421056	0.00000000
O	-2.73541962	-1.85709827	1.37705490
O	-0.02624625	1.91740084	1.24707126
O	-2.74740560	1.84632404	1.33704775
O	-1.58544249	3.91653292	0.00000000
O	2.73541962	1.85709827	1.37705490
O	1.57793476	4.07421056	0.00000000
O	-1.39729073	-0.00357424	2.62887778
O	1.39729073	0.00357424	2.62887778
O	4.24544798	-0.03767488	2.74310516
O	-4.24544798	0.03767488	2.74310516

[1,5] - $[Mo_2V_8O_{28}]^{4-}$ Symmol = NOSYM

Mo	0.01087669	2.35642213	1.57924628
V	0.00234113	2.27297467	-1.61323639
V	0.06467726	-2.30630691	1.47986842
V	-0.02857982	-2.26988100	-1.58309737
Mo	-1.59682662	-0.08894516	3.14506512
V	1.63353435	-0.06940135	3.07349556
V	-1.64101522	-0.00688284	-0.01606045
V	1.63694826	0.00630479	-0.04424173
V	-1.57943445	0.00346210	-3.16013863
V	1.56925088	-0.02509587	-3.14662867
O	2.61006070	0.01307670	1.41499022
O	2.67638264	-0.00075586	-1.37045363
O	1.26035926	-1.89185193	0.04009272
O	1.25612670	1.92066254	0.00798562
O	-0.04503299	0.03855693	1.39166014
O	0.02113257	-0.00331390	-1.22307220
O	1.31401656	-1.84871554	2.75424747
O	1.35478331	1.86529556	2.76219683
O	2.79063655	-0.05061488	4.19881461
O	0.02054184	0.02869721	4.10441634
O	1.33555278	-1.82307552	-2.69944062
O	1.34420141	1.79391918	-2.72812942
O	-0.00013512	-0.01955017	-4.09326551
O	2.74620588	-0.03723421	-4.25791986
O	-1.23501947	-1.88321586	0.02714369
O	0.04513776	-3.92107335	1.51140367
O	-1.34572525	-1.89945836	2.73367053
O	-0.02344433	-3.89346019	-1.56563867
O	-1.34548151	-1.81683837	-2.72266717
O	-1.24294528	1.91504284	-0.01013544
O	-1.33248077	1.80970761	-2.74493159
O	0.01266756	3.89716894	-1.61840582
O	-1.40351334	1.89317716	2.74835934
O	0.00761054	4.08485575	1.56909211
O	-2.62819211	0.00373885	-1.39643454
O	-2.67416209	0.01249757	1.39379552
O	-2.84337758	-0.05986224	4.33704830
O	-2.74767867	-0.00002698	-4.27869478

[1,6] - $[Mo_2V_8O_{28}]^{4-}$ Symmol = NOSYM

Mo	-0.01088794	2.35641801	1.57925339
V	-0.00233601	2.27297361	-1.61323150
V	-0.06465609	-2.30631068	1.47987507
V	0.02863299	-2.26987761	-1.58309475
V	-1.63350452	-0.06942066	3.07349047
Mo	1.59682390	-0.08894857	3.14506685
V	-1.63693662	0.00627553	-0.04417001
V	1.64100037	-0.00683984	-0.01616336
V	-1.56929061	-0.02512445	-3.14657213
V	1.57939059	0.00348503	-3.16022170
O	2.67404865	0.01247360	1.39383083
O	2.62829390	0.00374012	-1.39641882
O	1.23502259	-1.88320281	0.02716776
O	1.24299464	1.91501709	-0.01012214
O	0.04489135	0.03855386	1.39168689
O	-0.02106857	-0.00332600	-1.22305764
O	1.34568569	-1.89948473	2.73371365
O	1.40351238	1.89315843	2.74836091
O	2.84335567	-0.05987019	4.33706589
O	-0.02056037	0.02871430	4.10441832
O	1.34551522	-1.81679960	-2.72266779
O	1.33250239	1.80969006	-2.74494098
O	0.00009229	-0.01953709	-4.09327524
O	2.74761030	-0.00000338	-4.27880585
O	-1.26035608	-1.89186789	0.04008017
O	-0.04513357	-3.92107686	1.51140718
O	-1.31406226	-1.84872176	2.75422601
O	0.02350840	-3.89345590	-1.56564294
O	-1.33551713	-1.82308872	-2.69944409
O	-1.25607125	1.92068491	0.00796415
O	-1.34417586	1.79391171	-2.72809463
O	-0.01263494	3.89716784	-1.61841631
O	-1.35480835	1.86533802	2.76218317
O	-0.00757747	4.08485388	1.56908240
O	-2.67631396	-0.00075189	-1.37045586
O	-2.61011177	0.01311518	1.41495994
O	-2.79061689	-0.05061138	4.19880456
O	-2.74626105	-0.03725120	-4.25784189

[1,7] - $[Mo_2V_8O_{28}]^{4-}$ Symmol = NOSYM

Mo	0.02239975	2.33797976	1.60748584
V	-0.00372331	2.26369293	-1.63500488
V	0.00831652	-2.28894961	1.57907759
V	0.01576443	-2.26736291	-1.60985569
V	-1.52244942	-0.05062540	3.21429926
V	1.62791322	-0.07596666	3.12673937
Mo	-1.75748054	-0.01997616	-0.01351262
V	1.61027788	-0.02219075	0.00164417
V	-1.51868668	-0.01403968	-3.23725039
V	1.62146134	0.00219882	-3.13647692
O	2.61095598	0.00559779	1.40153247
O	2.61551553	0.00220412	-1.37522680
O	1.19066586	-1.88362890	0.01967992
O	1.20501551	1.91156155	-0.00639531
O	-0.04064864	0.03093019	1.33296636
O	-0.05153768	0.00708236	-1.27843886
O	1.34754240	-1.85627439	2.74430720
O	1.38949128	1.85137504	2.75698872
O	2.81009305	-0.06608370	4.22527961
O	0.06433638	0.01410964	4.10670481
O	1.35516373	-1.82143850	-2.72288072
O	1.35026915	1.81867293	-2.74084059
O	0.06431625	-0.01122314	-4.12996927
O	2.81644986	-0.00101838	-4.22420272
O	-1.29709175	-1.90702637	0.01884630
O	-0.02339856	-3.90597203	1.57568483
O	-1.31807753	-1.82484646	2.76784898
O	-0.00942914	-3.88975105	-1.59058309
O	-1.32568292	-1.80800570	-2.75135731
O	-1.30427620	1.92829950	-0.00251295
O	-1.33253670	1.79511993	-2.77532949
O	-0.02967768	3.88771782	-1.61885122
O	-1.35500558	1.83782825	2.77364316
O	-0.01099788	4.06629753	1.59962454
O	-2.76915626	0.00468770	-1.48448947
O	-2.72992479	0.00791276	1.49937872
O	-2.66099042	-0.03720986	4.36218736
O	-2.66517643	-0.02167897	-4.38074093

[1,8] - $[Mo_2V_8O_{28}]^{4-}$ Symmol = NOSYM

Mo	-0.02240767	2.33798023	1.60749840
V	0.00373406	2.26368695	-1.63500077
V	-0.00833232	-2.28895047	1.57907882
V	-0.01573650	-2.26735511	-1.60985490
V	-1.62791760	-0.07596051	3.12672310
V	1.52243704	-0.05064094	3.21431161
V	-1.61023978	-0.02219171	0.00163667
Mo	1.75751854	-0.01996233	-0.01351445
V	-1.62147499	0.00218637	-3.13643912
V	1.51867530	-0.01402827	-3.23730678
O	2.72993294	0.00790956	1.49940093
O	2.76917966	0.00466858	-1.48450219
O	1.29709294	-1.90698006	0.01885824
O	1.30429411	1.92826627	-0.00250786
O	0.04063055	0.03093417	1.33298674
O	0.05153853	0.00707810	-1.27844915
O	1.31806100	-1.82485286	2.76787037
O	1.35497816	1.83782207	2.77367257
O	2.66097567	-0.03722458	4.36220178
O	-0.06435946	0.01412911	4.10671682
O	1.32568033	-1.80798878	-2.75138872
O	1.33253869	1.79510222	-2.77535218
O	-0.06434688	-0.01121488	-4.12998490
O	2.66514918	-0.02166449	-4.38081398
O	-1.19065235	-1.88365405	0.01967598
O	0.02339713	-3.90597343	1.57567710
O	-1.34754756	-1.85627893	2.74430597
O	0.00947775	-3.88974456	-1.59058043
O	-1.35516460	-1.82143933	-2.72284901
O	-1.20498321	1.91158465	-0.00640739
O	-1.35027180	1.81866641	-2.74080285
O	0.02971354	3.88771160	-1.61885296
O	-1.38953852	1.85140410	2.75695438
O	0.01102355	4.06629813	1.59962544
O	-2.61553048	0.00220398	-1.37519961
O	-2.61092872	0.00558127	1.40151657
O	-2.81011727	-0.06608014	4.22524288
O	-2.81647897	-0.00102834	-4.22414711

[1,9] - $[Mo_2V_8O_{28}]^{4-}$ Symmol = NOSYM

Mo	-0.02316993	2.34435526	1.58478362
V	0.05303596	2.28862364	-1.56963076
V	-0.02136215	-2.29767505	1.55009574
V	0.06013208	-2.28156465	-1.54303208
V	-1.58544337	-0.06768293	3.14542423
V	1.57335541	-0.06512846	3.11612477
V	-1.64108584	-0.02975124	0.01597353
V	1.63013562	-0.01681390	0.01177852
Mo	-1.59488466	-0.00901270	-3.14390096
V	1.62810806	-0.00260399	-3.10154132
O	2.64066676	0.00382936	1.38308614
O	2.62535731	0.00243438	-1.40084915
O	1.24031720	-1.89450216	0.00168990
O	1.25398543	1.92516162	-0.02942058
O	0.00898415	0.03577172	1.28349735
O	-0.03684397	-0.00138905	-1.32532940
O	1.32683608	-1.84166459	2.71708688
O	1.36906762	1.84139667	2.72312520
O	2.74246716	-0.05281173	4.23322809
O	0.00081848	0.01412993	4.06512744
O	1.33721260	-1.83187185	-2.73245710
O	1.33497969	1.82196024	-2.75437399
O	0.02978411	-0.01400730	-4.11660309
O	2.79654371	-0.00854422	-4.21605745
O	-1.24276567	-1.88725734	0.02396520
O	-0.01463291	-3.91622713	1.55795719
O	-1.33497262	-1.84669834	2.74547908
O	0.03488936	-3.90214497	-1.54444052
O	-1.35235171	-1.86303595	-2.70563765
O	-1.24501894	1.92136170	-0.00868982
O	-1.35011472	1.85241568	-2.73014550
O	0.03299038	3.90983587	-1.58801765
O	-1.37504065	1.84830357	2.75844945
O	-0.00743364	4.07470642	1.59011397
O	-2.68346235	0.00657008	-1.36742375
O	-2.61969708	0.01066733	1.42254919
O	-2.74096364	-0.05629880	4.27506999
O	-2.85042329	-0.01483713	-4.32705473

[1,10] - $[Mo_2V_8O_{28}]^{4-}$ Symmol = NOSYM

Mo	0.02315312	2.34436125	1.58479826
V	-0.05301308	2.28861599	-1.56964396
V	0.02140325	-2.29767580	1.55007817
V	-0.06010645	-2.28155934	-1.54301781
V	-1.57337799	-0.06516069	3.11609607
V	1.58540328	-0.06767546	3.14545316
V	-1.63011550	-0.01681927	0.01177437
V	1.64109530	-0.02969009	0.01599456
V	-1.62811431	-0.00261306	-3.10152687
Mo	1.59488375	-0.00902549	-3.14392427
O	2.61971268	0.01064624	1.42255520
O	2.68340712	0.00656934	-1.36745817
O	1.24274837	-1.88721761	0.02394234
O	1.24506164	1.92133079	-0.00868053
O	-0.00897382	0.03577065	1.28351175
O	0.03681830	-0.00137250	-1.32532697
O	1.33497807	-1.84669203	2.74548747
O	1.37501989	1.84829264	2.75847754
O	2.74091236	-0.05628857	4.27510956
O	-0.00085876	0.01413543	4.06514689
O	1.35234290	-1.86306661	-2.70566186
O	1.35012465	1.85240559	-2.73020578
O	-0.02980768	-0.01401891	-4.11659741
O	2.85039184	-0.01487140	-4.32710811
O	-1.24032730	-1.89451689	0.00170557
O	0.01477130	-3.91621921	1.55793884
O	-1.32682529	-1.84167919	2.71707659
O	-0.03489941	-3.90213967	-1.54443211
O	-1.33723280	-1.83185261	-2.73241512
O	-1.25394614	1.92518216	-0.02940872
O	-1.33496252	1.82196878	-2.75436321
O	-0.03293004	3.90982871	-1.58801906
O	-1.36908354	1.84140786	2.72310878
O	0.00745877	4.07471210	1.59012852
O	-2.62536984	0.00246982	-1.40081281
O	-2.64066818	0.00384224	1.38307455
O	-2.74251178	-0.05284637	4.23317660
O	-2.79656216	-0.00853882	-4.21603205

[5,6] - $[Mo_2V_8O_{28}]^{4-}$ Symmol = C(2V)

V	-0.00000000	2.29134200	1.48710237
V	-0.00000000	2.26906715	-1.58478591
V	0.00000000	-2.29134200	1.48710237
V	0.00000000	-2.26906715	-1.58478591
Mo	-1.64173902	-0.00000000	3.12088207
Mo	1.64173902	0.00000000	3.12088207
V	-1.63794690	-0.00000000	-0.04215553
V	1.63794690	0.00000000	-0.04215553
V	-1.57234897	-0.00000000	-3.15792995
V	1.57234897	0.00000000	-3.15792995
O	2.67322703	0.00000000	1.38931635
O	2.65125976	0.00000000	-1.39007859
O	1.24523757	-1.88828890	0.02030953
O	1.24523757	1.88828890	0.02030953
O	0.00000000	0.00000000	1.41060919
O	0.00000000	0.00000000	-1.22330155
O	1.33848071	-1.86048537	2.73844590
O	1.33848071	1.86048537	2.73844590
O	2.89051157	0.00000000	4.30898534
O	0.00000000	0.00000000	4.16417769
O	1.33631158	-1.80769594	-2.72038522
O	1.33631158	1.80769594	-2.72038522
O	0.00000000	-0.00000000	-4.10001427
O	2.74575168	0.00000000	-4.27212666
O	-1.24523757	-1.88828890	0.02030953
O	0.00000000	-3.90862061	1.52021818
O	-1.33848071	-1.86048537	2.73844590
O	0.00000000	-3.89281790	-1.58190361
O	-1.33631158	-1.80769594	-2.72038522
O	-1.24523757	1.88828890	0.02030953
O	-1.33631158	1.80769594	-2.72038522
O	-0.00000000	3.89281790	-1.58190361
O	-1.33848071	1.86048537	2.73844590
O	-0.00000000	3.90862061	1.52021818
O	-2.65125976	-0.00000000	-1.39007859
O	-2.67322703	-0.00000000	1.38931635
O	-2.89051157	-0.00000000	4.30898534
O	-2.74575168	-0.00000000	-4.27212666

[5,7] - $[Mo_2V_8O_{28}]^{4-}$ Symmol = C(S)

V	0.06308021	1.56411396	-2.27215506
V	-0.01036797	-1.60583531	-2.26170412
V	0.06308021	1.56411396	2.27215506
V	-0.01036797	-1.60583531	2.26170412
Mo	-1.52892923	3.24602615	0.00000000
V	1.67933688	3.09230711	0.00000000
Mo	-1.76662922	-0.01062864	0.00000000
V	1.61101678	-0.03333887	0.00000000
V	-1.52924750	-3.25794294	0.00000000
V	1.61199053	-3.13770866	0.00000000
O	2.60480329	1.39619687	0.00000000
O	2.64859084	-1.36790631	0.00000000
O	1.20197367	0.02519317	1.88363906
O	1.20197367	0.02519317	-1.88363906
O	-0.09436142	1.37359628	0.00000000
O	-0.02379353	-1.25459798	0.00000000
O	1.34080153	2.75401962	1.82441541
O	1.34080153	2.75401962	-1.82441541
O	2.86763080	4.18201792	0.00000000
O	0.09147625	4.17626439	0.00000000
O	1.34822458	-2.71357823	1.81026578
O	1.34822458	-2.71357823	-1.81026578
O	0.06260463	-4.13651048	0.00000000
O	2.81033248	-4.22240192	0.00000000
O	-1.29387721	-0.00594087	1.89964341
O	0.01275429	1.57104864	3.89125921
O	-1.34348163	2.75350279	1.84190996
O	-0.02694102	-1.59347245	3.88446523
O	-1.32447050	-2.77092224	1.79550303
O	-1.29387721	-0.00594087	-1.89964341
O	-1.32447050	-2.77092224	-1.79550303
O	-0.02694102	-1.59347245	-3.88446523
O	-1.34348163	2.75350279	-1.84190996
O	0.01275429	1.57104864	-3.89125921
O	-2.74374363	-1.50857405	0.00000000
O	-2.79774144	1.47493732	0.00000000
O	-2.77415341	4.44243118	0.00000000
O	-2.66457501	-4.41042553	0.00000000

[5,8] - $[Mo_2V_8O_{28}]^{4-}$ Symmol = C(S)

V	0.03297039	1.56129908	-2.27647015
V	-0.02685430	-1.60473957	-2.26399648
V	0.03297039	1.56129908	2.27647015
V	-0.02685430	-1.60473957	2.26399648
Mo	-1.63619843	3.14905986	0.00000000
V	1.57446266	3.18905213	0.00000000
V	-1.61855494	0.00031663	0.00000000
Mo	1.75194716	-0.03628192	0.00000000
V	-1.62867835	-3.15639060	0.00000000
V	1.51090296	-3.23801262	0.00000000
O	2.70714469	1.50271990	0.00000000
O	2.81162114	-1.45996039	0.00000000
O	1.30054174	0.02566572	1.91008353
O	1.30054174	0.02566572	-1.91008353
O	-0.01319840	1.36207221	0.00000000
O	0.06806223	-1.26134560	0.00000000
O	1.31436501	2.76854079	1.80885906
O	1.31436501	2.76854079	-1.80885906
O	2.71914420	4.32806386	0.00000000
O	-0.02920110	4.16156279	0.00000000
O	1.32554402	-2.74168858	1.78911932
O	1.32554402	-2.74168858	-1.78911932
O	-0.06374666	-4.13940860	0.00000000
O	2.66757374	-4.37272875	0.00000000
O	-1.19144932	0.00038243	1.87925027
O	0.04147064	1.56712260	3.89580416
O	-1.36526938	2.72792834	1.85994043
O	0.00837294	-1.59678289	3.88661055
O	-1.34908511	-2.74229265	1.81597680
O	-1.19144932	0.00038243	-1.87925027
O	-1.34908511	-2.74229265	-1.81597680
O	0.00837294	-1.59678289	-3.88661055
O	-1.36526938	2.72792834	-1.85994043
O	0.04147064	1.56712260	-3.89580416
O	-2.59715597	-1.39760300	0.00000000
O	-2.68140937	1.37331443	0.00000000
O	-2.90555014	4.31351555	0.00000000
O	-2.81837869	-4.24881640	0.00000000

[5,9] - $[Mo_2V_8O_{28}]^{4-}$ Symmol = C(2V)

V	1.53518610	2.28127747	-0.03121130
V	-1.53518610	2.28127747	-0.03121130
V	1.53518610	-2.28127747	-0.03121130
V	-1.53518610	-2.28127747	-0.03121130
Mo	3.16666802	0.00000000	1.59879373
V	3.09203900	0.00000000	-1.62171339
V	0.00000000	-0.00000000	1.64491471
V	0.00000000	-0.00000000	-1.62609264
Mo	-3.16666802	-0.00000000	1.59879373
V	-3.09203900	-0.00000000	-1.62171339
O	1.38514789	0.00000000	-2.63916345
O	-1.38514789	-0.00000000	-2.63916345
O	0.00000000	-1.89834901	-1.24847258
O	-0.00000000	1.89834901	-1.24847258
O	1.31449566	0.00000000	0.02517999
O	-1.31449566	-0.00000000	0.02517999
O	2.72069571	-1.81710199	-1.32773233
O	2.72069571	1.81710199	-1.32773233
O	4.20130061	0.00000000	-2.79632517
O	4.12240258	0.00000000	-0.03146893
O	-2.72069571	-1.81710199	-1.32773233
O	-2.72069571	1.81710199	-1.32773233
O	-4.12240258	-0.00000000	-0.03146893
O	-4.20130061	-0.00000000	-2.79632517
O	0.00000000	-1.88276273	1.23620468
O	1.55231626	-3.90217015	-0.01837586
O	2.72831380	-1.85371335	1.35174838
O	-1.55231626	-3.90217015	-0.01837586
O	-2.72831380	-1.85371335	1.35174838
O	-0.00000000	1.88276273	1.23620468
O	-2.72831380	1.85371335	1.35174838
O	-1.55231626	3.90217015	-0.01837586
O	2.72831380	1.85371335	1.35174838
O	1.55231626	3.90217015	-0.01837586
O	-1.39175564	-0.00000000	2.67646084
O	1.39175564	0.00000000	2.67646084
O	4.36094268	0.00000000	2.84223546
O	-4.36094268	-0.00000000	2.84223546

[5,10] - $[Mo_2V_8O_{28}]^{4-}$ Symmol = C(2H)

V	0.08338970	-1.53807597	2.27933010
V	-0.08338970	1.53807597	2.27933010
V	0.08338970	-1.53807597	-2.27933010
V	-0.08338970	1.53807597	-2.27933010
Mo	-1.59218968	-3.14091993	0.00000000
V	1.62915781	-3.12843042	0.00000000
V	-1.63884767	0.00329056	0.00000000
V	1.63884767	-0.00329056	0.00000000
V	-1.62915781	3.12843042	0.00000000
Mo	1.59218968	3.14091993	0.00000000
O	2.61824276	-1.42404484	0.00000000
O	2.69586416	1.35541269	0.00000000
O	1.24556305	-0.01942026	-1.89159042
O	1.24556305	-0.01942026	1.89159042
O	-0.04469489	-1.31048318	0.00000000
O	0.04469489	1.31048318	0.00000000
O	1.33588924	-2.75500421	-1.82209602
O	1.33588924	-2.75500421	1.82209602
O	2.79349876	-4.24628627	0.00000000
O	0.02702868	-4.12670233	0.00000000
O	1.34323365	2.70191918	-1.84704450
O	1.34323365	2.70191918	1.84704450
O	-0.02702868	4.12670233	0.00000000
O	2.85537879	4.31658317	0.00000000
O	-1.24556305	0.01942026	-1.89159042
O	0.04904423	-1.55507159	-3.89997366
O	-1.34323365	-2.70191918	-1.84704450
O	-0.04904423	1.55507159	-3.89997366
O	-1.33588924	2.75500421	-1.82209602
O	-1.24556305	0.01942026	1.89159042
O	-1.33588924	2.75500421	1.82209602
O	-0.04904423	1.55507159	3.89997366
O	-1.34323365	-2.70191918	1.84704450
O	0.04904423	-1.55507159	3.89997366
O	-2.61824276	1.42404484	0.00000000
O	-2.69586416	-1.35541269	0.00000000
O	-2.85537879	-4.31658317	0.00000000
O	-2.79349876	4.24628627	0.00000000

[7,8] - $[Mo_2V_8O_{28}]^{4-}$ Symmol = D(2H)

V	-0.00000000	2.25939997	1.63125056
V	-0.00000000	2.25939997	-1.63125056
V	0.00000000	-2.25939997	1.63125056
V	0.00000000	-2.25939997	-1.63125056
V	-1.56709213	-0.00000000	3.23408771
V	1.56709213	0.00000000	3.23408771
Mo	-1.72751087	0.00000000	0.00000000
Mo	1.72751087	-0.00000000	0.00000000
V	-1.56709213	-0.00000000	-3.23408771
V	1.56709213	0.00000000	-3.23408771
O	2.72754178	0.00000000	1.48566891
O	2.72754178	0.00000000	-1.48566891
O	1.24802833	-1.89938936	0.00000000
O	1.24802833	1.89938936	0.00000000
O	0.00000000	0.00000000	1.30874322
O	-0.00000000	0.00000000	-1.30874322
O	1.33722238	-1.80347841	2.76707011
O	1.33722238	1.80347841	2.76707011
O	2.73253631	0.00000000	4.35316919
O	0.00000000	0.00000000	4.17380306
O	1.33722238	-1.80347841	-2.76707011
O	1.33722238	1.80347841	-2.76707011
O	-0.00000000	0.00000000	-4.17380306
O	2.73253631	0.00000000	-4.35316919
O	-1.24802833	-1.89938936	0.00000000
O	0.00000000	-3.88108160	1.61314628
O	-1.33722238	-1.80347841	2.76707011
O	0.00000000	-3.88108160	-1.61314628
O	-1.33722238	-1.80347841	-2.76707011
O	-1.24802833	1.89938936	0.00000000
O	-1.33722238	1.80347841	-2.76707011
O	-0.00000000	3.88108160	-1.61314628
O	-1.33722238	1.80347841	2.76707011
O	-0.00000000	3.88108160	1.61314628
O	-2.72754178	-0.00000000	-1.48566891
O	-2.72754178	-0.00000000	1.48566891
O	-2.73253631	-0.00000000	4.35316919
O	-2.73253631	-0.00000000	-4.35316919

[1,2,3] - $[Mo_3V_7O_{28}]^{3-}$, Symmol = C(S)

Mo	1.61257771	2.36556200	0.00000000
Mo	-1.63511308	2.34664441	0.00000000
Mo	1.57028783	-2.36468187	0.00000000
V	-1.59953130	-2.30059069	0.00000000
V	3.13013241	-0.00936975	1.59573229
V	3.13013241	-0.00936975	-1.59573229
V	-0.00653031	-0.01505289	1.63828284
V	-0.00653031	-0.01505289	-1.63828284
V	-3.14139603	-0.06788592	1.58545051
V	-3.14139603	-0.06788592	-1.58545051
O	1.40999744	0.00722636	-2.61366007
O	-1.39612932	0.00822691	-2.62389226
O	-0.01008763	-1.91004878	-1.24725122
O	0.01670630	1.94880060	-1.25419439
O	1.31385771	0.00870780	0.00000000
O	-1.27599328	0.03577435	0.00000000
O	2.74697578	-1.87070168	-1.36883211
O	2.77608386	1.86174529	-1.37169751
O	4.25613111	-0.01694939	-2.74217222
O	4.04402154	-0.01286795	0.00000000
O	-2.74329110	-1.85065673	-1.33912429
O	-2.75641875	1.84810319	-1.38138713
O	-4.06303628	0.00945877	0.00000000
O	-4.26136115	-0.05674215	-2.74241507
O	-0.01008763	-1.91004878	1.24725122
O	1.56940807	-4.08269596	0.00000000
O	2.74697578	-1.87070168	1.36883211
O	-1.57905697	-3.91450721	0.00000000
O	-2.74329110	-1.85065673	1.33912429
O	0.01670630	1.94880060	1.25419439
O	-2.75641875	1.84810319	1.38138713
O	-1.62000100	4.07075524	0.00000000
O	2.77608386	1.86174529	1.37169751
O	1.62095379	4.08505098	0.00000000
O	-1.39612932	0.00822691	2.62389226
O	1.40999744	0.00722636	2.61366007
O	4.25613111	-0.01694939	2.74217222
O	-4.26136115	-0.05674215	2.74241507

[1,2,5] - $[Mo_3V_7O_{28}]^{3-}$ Symmol = NOSYM

Mo	0.01656689	2.36300123	1.61691212
Mo	-0.00688823	2.34943346	-1.62989384
V	0.06709539	-2.31135825	1.51159221
V	-0.02872502	-2.29851621	-1.56445384
Mo	-1.60942929	-0.09233390	3.14736580
V	1.63606330	-0.06563981	3.09594984
V	-1.63940340	-0.03612721	-0.02410550
V	1.63315825	-0.01898188	-0.01855032
V	-1.58691889	-0.06663490	-3.16428942
V	1.58504037	-0.08631079	-3.13635299
O	2.61331081	0.01545119	1.41081418
O	2.63611591	0.00526638	-1.38390829
O	1.24372561	-1.88652916	0.01470551
O	1.25844926	1.95191123	0.01658838
O	-0.03287077	0.04340232	1.35362811
O	0.00536503	0.03606881	-1.24826574
O	1.32786824	-1.85219204	2.74092876
O	1.36675504	1.85467124	2.77786130
O	2.78968083	-0.05997823	4.21454416
O	0.02220568	0.00701588	4.09385619
O	1.32507583	-1.85186523	-2.71296770
O	1.37629027	1.82423802	-2.74673239
O	0.00300422	-0.00488618	-4.06736154
O	2.74609933	-0.08978364	-4.25172057
O	-1.23907086	-1.87894559	0.00662205
O	0.05266610	-3.92039813	1.51520544
O	-1.34316803	-1.90324258	2.72089886
O	-0.02847179	-3.91118694	-1.55006439
O	-1.34304429	-1.84491020	-2.73461647
O	-1.24917888	1.95465443	0.00214030
O	-1.37276854	1.83954915	-2.77029919
O	0.01011036	4.07328759	-1.64158080
O	-1.39589043	1.88283551	2.76748924
O	0.01749143	4.08549834	1.61209384
O	-2.61714437	0.01658181	-1.41310120
O	-2.65971718	0.02144479	1.38886853
O	-2.84298984	-0.08204630	4.34068079
O	-2.73645835	-0.06244419	-4.29048142

[1,2,6] - $[Mo_3V_7O_{28}]^{3-}$ Symmol = NOSYM

Mo	-0.01655398	2.36299899	1.61691663
Mo	0.00686776	2.34943794	-1.62989103
V	-0.06707454	-2.31136428	1.51158470
V	0.02872044	-2.29852026	-1.56445219
V	-1.63605574	-0.06565321	3.09592769
Mo	1.60942532	-0.09234066	3.14738468
V	-1.63314350	-0.01900182	-0.01856777
V	1.63941328	-0.03609116	-0.02410033
V	-1.58505607	-0.08633543	-3.13636674
V	1.58690101	-0.06661010	-3.16426761
O	2.65970694	0.02141823	1.38889687
O	2.61715387	0.01657193	-1.41309644
O	1.23905980	-1.87891360	0.00662563
O	1.24921960	1.95462074	0.00214969
O	0.03283827	0.04340885	1.35363865
O	-0.00538573	0.03606276	-1.24826257
O	1.34317490	-1.90325814	2.72091431
O	1.39588924	1.88282099	2.76750903
O	2.84297734	-0.08204142	4.34070807
O	-0.02222250	0.00702635	4.09384702
O	1.34300625	-1.84491130	-2.73465176
O	1.37275894	1.83953761	-2.77030862
O	-0.00303649	-0.00488714	-4.06735837
O	2.73642870	-0.06242507	-4.29047037
O	-1.24373992	-1.88656224	0.01470741
O	-0.05264693	-3.92040275	1.51520622
O	-1.32787013	-1.85219274	2.74091900
O	0.02858406	-3.91118632	-1.55006073
O	-1.32511330	-1.85187173	-2.71293493
O	-1.25840673	1.95195086	0.01656894
O	-1.37629494	1.82424835	-2.74672230
O	-0.01009024	4.07329184	-1.64158674
O	-1.36675016	1.85469007	2.77784231
O	-0.01745238	4.08549829	1.61209821
O	-2.63611664	0.00528957	-1.38391484
O	-2.61331068	0.01548218	1.41077843
O	-2.78968122	-0.05998046	4.21451567
O	-2.74612388	-0.08980574	-4.25172585

[1,2,7] - $[Mo_3V_7O_{28}]^{3-}$ Symmol = C(S)

Mo	0.00714494	2.34232109	1.64960678
Mo	0.00714494	2.34232109	-1.64960678
V	0.01346094	-2.29536019	1.59511564
V	0.01346094	-2.29536019	-1.59511564
V	-1.53289640	-0.06515090	3.23318941
V	1.63231239	-0.07451281	3.13996312
Mo	-1.75622464	-0.03990686	0.00000000
V	1.61090509	-0.03775894	0.00000000
V	-1.53289640	-0.06515090	-3.23318941
V	1.63231239	-0.07451281	-3.13996312
O	2.60001117	0.00956503	1.39502362
O	2.60001117	0.00956503	-1.39502362
O	1.18679199	-1.88126519	0.00000000
O	1.21424725	1.94315204	0.00000000
O	-0.04638996	0.03799668	1.29777280
O	-0.04638996	0.03799668	-1.29777280
O	1.35017208	-1.85552513	2.73724466
O	1.38748645	1.84525466	2.76701319
O	2.80974298	-0.07087124	4.23403080
O	0.06265822	0.00211349	4.10354086
O	1.35017208	-1.85552513	-2.73724466
O	1.38748645	1.84525466	-2.76701319
O	0.06265822	0.00211349	-4.10354086
O	2.80974298	-0.07087124	-4.23403080
O	-1.29398143	-1.90623975	0.00000000
O	-0.01551707	-3.90657088	1.57696682
O	-1.31905289	-1.83070370	2.76266756
O	-0.01551707	-3.90657088	-1.57696682
O	-1.31905289	-1.83070370	-2.76266756
O	-1.30392190	1.95393139	0.00000000
O	-1.36517722	1.82940841	-2.79330217
O	-0.02168610	4.06475740	-1.63696519
O	-1.36517722	1.82940841	2.79330217
O	-0.02168610	4.06475740	1.63696519
O	-2.73106998	0.01547931	-1.50066891
O	-2.73106998	0.01547931	1.50066891
O	-2.66010773	-0.06415756	4.38263511
O	-2.66010773	-0.06415756	-4.38263511

[1,3,5] - $[Mo_3V_7O_{28}]^{3-}$ Symmol = C(S)

Mo	0.03168163	1.54125963	-2.37698244
V	-0.01539979	-1.63127364	-2.27232869
Mo	0.03168163	1.54125963	2.37698244
V	-0.01539979	-1.63127364	2.27232869
Mo	-1.60918221	3.15642408	0.00000000
V	1.65414497	3.07984945	0.00000000
V	-1.64459468	-0.02088362	0.00000000
V	1.63890426	-0.04872913	0.00000000
V	-1.58316001	-3.18510550	0.00000000
V	1.57243261	-3.16816035	0.00000000
O	2.60167139	1.42999629	0.00000000
O	2.67124335	-1.36728334	0.00000000
O	1.26844058	0.03186034	1.91746130
O	1.26844058	0.03186034	-1.91746130
O	-0.04539555	1.40165629	0.00000000
O	0.02294554	-1.19904614	0.00000000
O	1.34091004	2.78276481	1.86919359
O	1.34091004	2.78276481	-1.86919359
O	2.79901245	4.20371191	0.00000000
O	0.02112830	4.09372423	0.00000000
O	1.34165249	-2.72306617	1.80509919
O	1.34165249	-2.72306617	-1.80509919
O	-0.00055685	-4.10352709	0.00000000
O	2.74584507	-4.27421921	0.00000000
O	-1.24413218	0.01038857	1.90928622
O	0.02358254	1.55460401	4.09319337
O	-1.38788091	2.75636079	1.90558715
O	-0.00567936	-1.60610394	3.89098611
O	-1.34209075	-2.74626880	1.81192636
O	-1.24413218	0.01038857	-1.90928622
O	-1.34209075	-2.74626880	-1.81192636
O	-0.00567936	-1.60610394	-3.89098611
O	-1.38788091	2.75636079	-1.90558715
O	0.02358254	1.55460401	-4.09319337
O	-2.62035332	-1.39751332	0.00000000
O	-2.66270615	1.40297524	0.00000000
O	-2.83692568	4.35467219	0.00000000
O	-2.74662207	-4.29959319	0.00000000

[1,3,7] - $[Mo_3V_7O_{28}]^{3-}$ Symmol = C(S)

Mo	0.01730492	1.59996374	-2.35435926
V	-0.00062845	-1.65152142	-2.26564056
Mo	0.01730492	1.59996374	2.35435926
V	-0.00062845	-1.65152142	2.26564056
V	-1.53649691	3.22315513	0.00000000
V	1.64348708	3.13601040	0.00000000
Mo	-1.76126164	-0.02332208	0.00000000
V	1.61601051	-0.00198941	0.00000000
V	-1.52339913	-3.26833656	0.00000000
V	1.62552342	-3.15750439	0.00000000
O	2.60024941	1.41683205	0.00000000
O	2.61027072	-1.37163618	0.00000000
O	1.20511935	0.01155463	1.90516753
O	1.20511935	0.01155463	-1.90516753
O	-0.03724033	1.35313568	0.00000000
O	-0.05263097	-1.24664900	0.00000000
O	1.38219310	2.77408278	1.86955143
O	1.38219310	2.77408278	-1.86955143
O	2.80991563	4.23709096	0.00000000
O	0.06231324	4.09508541	0.00000000
O	1.35154024	-2.73895037	1.81441810
O	1.35154024	-2.73895037	-1.81441810
O	0.06431461	-4.14213143	0.00000000
O	2.81818376	-4.23889520	0.00000000
O	-1.30456293	0.01400326	1.92626179
O	-0.01508350	1.59409938	4.07134943
O	-1.34968834	2.79083569	1.84841570
O	-0.02304917	-1.61933521	3.88368909
O	-1.33275750	-2.77557444	1.79674380
O	-1.30456293	0.01400326	-1.92626179
O	-1.33275750	-2.77557444	-1.79674380
O	-0.02304917	-1.61933521	-3.88368909
O	-1.34968834	2.79083569	-1.84841570
O	-0.01508350	1.59409938	-4.07134943
O	-2.77386834	-1.48235336	0.00000000
O	-2.70568764	1.51139135	0.00000000
O	-2.65895696	4.37298854	0.00000000
O	-2.66150193	-4.41118795	0.00000000

[1,3,9] - $[Mo_3V_7O_{28}]^{3-}$ Symmol = C(S)

Mo	-0.02325648	1.57193157	-2.36234377
V	0.05579903	-1.58808718	-2.28703263
Mo	-0.02325648	1.57193157	2.36234377
V	0.05579903	-1.58808718	2.28703263
V	-1.59347464	3.15579478	0.00000000
V	1.59197468	3.11929336	0.00000000
V	-1.64787755	0.01672578	0.00000000
V	1.63031222	-0.00802298	0.00000000
Mo	-1.60196274	-3.16167060	0.00000000
V	1.63247494	-3.12385026	0.00000000
O	2.61405737	1.39294612	0.00000000
O	2.62786518	-1.40023770	0.00000000
O	1.24812233	-0.00929119	1.91677542
O	1.24812233	-0.00929119	-1.91677542
O	-0.00570565	1.30524158	0.00000000
O	-0.02893078	-1.29272782	0.00000000
O	1.35962318	2.73946932	1.85707843
O	1.35962318	2.73946932	-1.85707843
O	2.74718239	4.23672012	0.00000000
O	0.00469064	4.05301117	0.00000000
O	1.34223311	-2.75014720	1.82416664
O	1.34223311	-2.75014720	-1.82416664
O	0.03032320	-4.11815790	0.00000000
O	2.79685305	-4.23312296	0.00000000
O	-1.25373107	0.01015607	1.91306279
O	-0.01297754	1.58141375	4.08145901
O	-1.36957368	2.77291481	1.86302797
O	0.03791655	-1.57798883	3.90269440
O	-1.34975814	-2.72562597	1.85487870
O	-1.25373107	0.01015607	-1.91306279
O	-1.34975814	-2.72562597	-1.85487870
O	0.03791655	-1.57798883	-3.90269440
O	-1.36957368	2.77291481	-1.86302797
O	-0.01297754	1.58141375	-4.08145901
O	-2.67266152	-1.36863087	0.00000000
O	-2.61424791	1.43412937	0.00000000
O	-2.73321239	4.28836041	0.00000000
O	-2.84645506	-4.34529192	0.00000000

[1,4,5] - $[Mo_3V_7O_{28}]^{3-}$ Symmol = NOSYM

Mo	0.01864585	2.35639770	1.57969899
V	-0.00317274	2.29890657	-1.60045503
V	0.05731234	-2.31377324	1.54389474
Mo	-0.02189392	-2.34355800	-1.59295727
Mo	-1.60672557	-0.07187460	3.15541915
V	1.63791946	-0.05797657	3.09440864
V	-1.64245897	0.01339685	-0.01600791
V	1.63561000	0.01283199	-0.02492569
V	-1.58817183	0.06453263	-3.16189203
V	1.58184444	0.04516099	-3.14201132
O	2.61045060	0.00467383	1.41184475
O	2.64261819	-0.00471618	-1.38462980
O	1.25600852	-1.91633227	0.04481787
O	1.24946856	1.91460205	-0.01093883
O	-0.03839518	0.03511342	1.35354387
O	0.01277039	-0.03066297	-1.25268754
O	1.32269451	-1.84446878	2.76781426
O	1.36223249	1.86244704	2.75339713
O	2.79186642	-0.03931295	4.21234900
O	0.02303425	0.03282864	4.09713933
O	1.37055157	-1.84786503	-2.71988142
O	1.33802636	1.82767105	-2.74174801
O	0.00217637	-0.02840569	-4.06919767
O	2.74242468	0.02406613	-4.25789412
O	-1.24270654	-1.90878652	0.03450192
O	0.04243146	-3.92401030	1.55740130
O	-1.34732752	-1.89104993	2.74785617
O	-0.01733159	-4.06663956	-1.57943876
O	-1.37900131	-1.84400631	-2.74560024
O	-1.24460352	1.91081299	-0.02807051
O	-1.33018621	1.84121079	-2.76147887
O	0.00631970	3.91290084	-1.59949685
O	-1.39928924	1.88982662	2.73864090
O	0.01450239	4.07762366	1.55880577
O	-2.61166285	-0.00095943	-1.41394493
O	-2.66603857	0.00566249	1.38942695
O	-2.84034190	-0.04820145	4.34855479
O	-2.73960105	0.05193350	-4.28625875

[1,4,6] - $[Mo_3V_7O_{28}]^{3-}$ Symmol = NOSYM

Mo	-0.01835246	2.35638726	1.57973085
V	0.00294066	2.29868410	-1.60046687
V	-0.05787484	-2.31353515	1.54382004
Mo	0.02229252	-2.34336645	-1.59301022
V	-1.63823650	-0.05771392	3.09454626
Mo	1.60661448	-0.07223452	3.15543951
V	-1.63573663	0.01294022	-0.02542386
V	1.64261213	0.01331189	-0.01540255
V	-1.58168018	0.04481294	-3.14217950
V	1.58833952	0.06485334	-3.16180612
O	2.66665942	0.00571529	1.38945549
O	2.61119994	-0.00098119	-1.41399434
O	1.24278815	-1.90874924	0.03423129
O	1.24454572	1.91077135	-0.02821155
O	0.03940322	0.03481565	1.35345871
O	-0.01318048	-0.03066118	-1.25288527
O	1.34732143	-1.89124322	2.74767968
O	1.39945400	1.88981602	2.73854715
O	2.84026537	-0.04849353	4.34857467
O	-0.02290818	0.03291119	4.09716966
O	1.37903325	-1.84396938	-2.74596646
O	1.33008716	1.84139776	-2.76159858
O	-0.00211981	-0.02836598	-4.06924560
O	2.73981576	0.05234459	-4.28611832
O	-1.25604699	-1.91622797	0.04492710
O	-0.04295422	-3.92377397	1.55739726
O	-1.32279452	-1.84417520	2.76812984
O	0.01760637	-4.06645152	-1.57920818
O	-1.37058205	-1.84797734	-2.71976901
O	-1.24949640	1.91444682	-0.01078018
O	-1.33821409	1.82749965	-2.74166118
O	-0.00673780	3.91269148	-1.59931478
O	-1.36206362	1.86236525	2.75338776
O	-0.01433944	4.07759675	1.55889121
O	-2.64318343	-0.00487779	-1.38459115
O	-2.61009611	0.00471115	1.41184346
O	-2.79213041	-0.03897941	4.21248829
O	-2.74225095	0.02370423	-4.25808453

[1,4,7] - $[Mo_3V_7O_{28}]^{3-}$ Symmol = C(2)

Mo	1.61828735	2.33893656	-0.02205218
V	-1.62249400	2.28996925	0.00583561
V	1.62249400	-2.28996925	0.00583561
Mo	-1.61828735	-2.33893656	-0.02205218
V	3.23747383	-0.03838845	1.52923914
V	3.14241993	-0.07533987	-1.63391345
Mo	0.00000000	-0.00000000	1.76205343
V	0.00000000	-0.00000000	-1.61320116
V	-3.23747383	0.03838845	1.52923914
V	-3.14241993	0.07533987	-1.63391345
O	1.39404755	0.00244483	-2.60243462
O	-1.39404755	-0.00244483	-2.60243462
O	0.02726197	-1.90567102	-1.20087482
O	-0.02726197	1.90567102	-1.20087482
O	1.30122021	0.02623842	0.04727933
O	-1.30122021	-0.02623842	0.04727933
O	2.75807075	-1.85077436	-1.34685290
O	2.74866163	1.84810911	-1.39142698
O	4.23512833	-0.06448603	-2.81303068
O	4.10804312	0.02346344	-0.06476826
O	-2.74866163	-1.84810911	-1.39142698
O	-2.75807075	1.85077436	-1.34685290
O	-4.10804312	-0.02346344	-0.06476826
O	-4.23512833	0.06448603	-2.81303068
O	0.02288225	-1.92382082	1.30139770
O	1.60609481	-3.90275917	0.03485169
O	2.78891331	-1.81773490	1.32485366
O	-1.59487949	-4.06039477	0.00719086
O	-2.76809769	-1.83872290	1.35829714
O	-0.02288225	1.92382082	1.30139770
O	-2.78891331	1.81773490	1.32485366
O	-1.60609481	3.90275917	0.03485169
O	2.76809769	1.83872290	1.35829714
O	1.59487949	4.06039477	0.00719086
O	-1.50111490	-0.00054878	2.73541481
O	1.50111490	0.00054878	2.73541481
O	4.38686234	-0.02213869	2.65656779
O	-4.38686234	0.02213869	2.65656779

[1,4,8] - $[Mo_3V_7O_{28}]^{3-}$ Symmol = C(2)

Mo	1.61828912	2.33892587	0.02204772
V	-1.62249139	2.28996443	-0.00582329
V	1.62249139	-2.28996443	-0.00582329
Mo	-1.61828912	-2.33892587	0.02204772
V	3.14242482	-0.07533476	1.63391372
V	3.23749186	-0.03839731	-1.52924075
V	-0.00000000	0.00000000	1.61320542
Mo	-0.00000000	0.00000000	-1.76205583
V	-3.14242482	0.07533476	1.63391372
V	-3.23749186	0.03839731	-1.52924075
O	1.50110998	0.00057016	-2.73542114
O	-1.50110998	-0.00057016	-2.73542114
O	0.02288668	-1.92381682	-1.30139206
O	-0.02288668	1.92381682	-1.30139206
O	1.30121354	0.02623747	-0.04726618
O	-1.30121354	-0.02623747	-0.04726618
O	2.78891612	-1.81773214	-1.32485551
O	2.76810334	1.83871202	-1.35829272
O	4.38687458	-0.02215134	-2.65657439
O	4.10804820	0.02346465	0.06476779
O	-2.76810334	-1.83871202	-1.35829272
O	-2.78891612	1.81773214	-1.32485551
O	-4.10804820	-0.02346465	0.06476779
O	-4.38687458	0.02215134	-2.65657439
O	0.02726429	-1.90567389	1.20088109
O	1.60609231	-3.90275321	-0.03484860
O	2.75808166	-1.85077141	1.34685049
O	-1.59486579	-4.06038251	-0.00721605
O	-2.74866457	-1.84810992	1.39143005
O	-0.02726429	1.90567389	1.20088109
O	-2.75808166	1.85077141	1.34685049
O	-1.60609231	3.90275321	-0.03484860
O	2.74866457	1.84810992	1.39143005
O	1.59486579	4.06038251	-0.00721605
O	-1.39405322	-0.00243751	2.60243628
O	1.39405322	0.00243751	2.60243628
O	4.23513520	-0.06448271	2.81302877
O	-4.23513520	0.06448271	2.81302877

[1,5,6] - $[Mo_3V_7O_{28}]^{3-}$ Symmol = C(S)

Mo	1.56822103	2.37164400	0.00000000
V	-1.63047617	2.27256063	0.00000000
V	1.43478039	-2.31890528	0.00000000
V	-1.60007219	-2.27100250	0.00000000
Mo	3.12487341	-0.08252932	1.65027255
Mo	3.12487341	-0.08252932	-1.65027255
V	-0.04790047	0.00960678	1.63977381
V	-0.04790047	0.00960678	-1.63977381
V	-3.18524599	-0.01307584	1.57683237
V	-3.18524599	-0.01307584	-1.57683237
O	1.40911834	0.01529508	-2.65570426
O	-1.38447568	0.00320533	-2.64859794
O	0.04631736	-1.88390859	-1.25400125
O	0.00785182	1.91132833	-1.24948891
O	1.44095013	0.03731506	0.00000000
O	-1.18682569	-0.00727229	0.00000000
O	2.76098967	-1.89194071	-1.32475945
O	2.76567322	1.89706201	-1.37878366
O	4.32005314	-0.05762389	-2.88046808
O	4.14527477	0.04181590	0.00000000
O	-2.71517095	-1.81194581	-1.33898300
O	-2.74261359	1.79029301	-1.33596884
O	-4.11193825	-0.02265482	0.00000000
O	-4.29510620	-0.02187489	-2.74541857
O	0.04631736	-1.88390859	1.25400125
O	1.46989706	-3.92474046	0.00000000
O	2.76098967	-1.89194071	1.32475945
O	-1.57544524	-3.88842568	0.00000000
O	-2.71517095	-1.81194581	1.33898300
O	0.00785182	1.91132833	1.24948891
O	-2.74261359	1.79029301	1.33596884
O	-1.63195316	3.89062933	0.00000000
O	2.76567322	1.89706201	1.37878366
O	1.54885920	4.09125312	0.00000000
O	-1.38447568	0.00320533	2.64859794
O	1.40911834	0.01529508	2.65570426
O	4.32005314	-0.05762389	2.88046808
O	-4.29510620	-0.02187489	2.74541857

[1,5,7] - $[Mo_3V_7O_{28}]^{3-}$ Symmol = NOSYM

Mo	0.05983411	2.34849990	1.60059524
V	-0.01947161	2.26498570	-1.65180640
V	0.04424096	-2.30012833	1.53752488
V	-0.00501443	-2.26258311	-1.61762000
Mo	-1.54067611	-0.04786194	3.25414181
V	1.68897709	-0.08927832	3.09480915
Mo	-1.76786639	-0.00016794	-0.02035429
V	1.61426331	-0.01611528	-0.03187654
V	-1.53096223	-0.01698505	-3.28554816
V	1.61925878	-0.00381294	-3.15871819
O	2.59515303	0.01625658	1.41339136
O	2.64000742	0.00469774	-1.36278583
O	1.21035020	-1.87827452	0.04432581
O	1.21019169	1.91268416	0.01438432
O	-0.08675239	0.03953525	1.39952086
O	-0.02632144	0.00290805	-1.22006485
O	1.33040483	-1.85649882	2.76188251
O	1.37342429	1.85499964	2.78933286
O	2.86556603	-0.08728513	4.18564540
O	0.08952269	0.01895612	4.15717021
O	1.35096628	-1.80875963	-2.71000897
O	1.34527836	1.80183333	-2.73246579
O	0.06552713	-0.01656893	-4.14682891
O	2.81496339	-0.00824886	-4.23711033
O	-1.28661714	-1.89322860	0.02269050
O	0.00457484	-3.90769334	1.54225156
O	-1.33562867	-1.86338965	2.76985476
O	-0.01992075	-3.87925100	-1.58803586
O	-1.32437718	-1.79887185	-2.76504641
O	-1.30183441	1.92416123	-0.00862545
O	-1.32970714	1.77943518	-2.79252158
O	-0.03770371	3.88286999	-1.63700721
O	-1.38047752	1.87285123	2.77577581
O	0.00916283	4.06822969	1.58951338
O	-2.74806147	-0.00028372	-1.50244815
O	-2.76685935	0.00606384	1.49180491
O	-2.76355421	-0.03592328	4.46024544
O	-2.65986107	-0.02775737	-4.43598786

[1,5,8] - $[Mo_3V_7O_{28}]^{3-}$ Symmol = NOSYM

Mo	-0.03019591	2.35294990	1.60703044
V	0.00718184	2.26775508	-1.65260163
V	0.08123218	-2.30397275	1.53030001
V	-0.03856856	-2.26631165	-1.61467352
Mo	-1.65653477	-0.11181237	3.15259428
V	1.57265089	-0.02916154	3.19180340
V	-1.61646701	-0.02543633	-0.01024952
Mo	1.75698138	0.01886790	-0.03708141
V	-1.62718708	0.00999379	-3.17422556
V	1.51944517	-0.03258828	-3.26640523
O	2.69421035	-0.00103539	1.51244002
O	2.80300654	-0.01189366	-1.46144153
O	1.29516563	-1.90391403	0.03778799
O	1.31419165	1.93283607	0.01251965
O	-0.00518550	0.03887657	1.38859780
O	0.05756370	0.00469790	-1.22706637
O	1.30930578	-1.82334767	2.78410777
O	1.34450734	1.84550619	2.78085883
O	2.70192817	-0.02090709	4.33390828
O	-0.03959184	0.01974606	4.14211391
O	1.31421664	-1.80512883	-2.74028079
O	1.34053732	1.76398097	-2.76980648
O	-0.06325409	-0.01819685	-4.14846846
O	2.66541378	-0.05378072	-4.40174797
O	-1.20101083	-1.86817581	0.02946024
O	0.08205708	-3.91209947	1.54026138
O	-1.35183807	-1.90859698	2.74039244
O	-0.02465260	-3.88309035	-1.58805864
O	-1.36264936	-1.80414969	-2.72959745
O	-1.19258835	1.91455241	-0.00334819
O	-1.33567773	1.81681565	-2.75601189
O	0.05184990	3.88517678	-1.64369372
O	-1.40928595	1.88687210	2.77853867
O	0.01841278	4.07234696	1.58444439
O	-2.59708411	0.01884263	-1.38679300
O	-2.65567812	0.02603319	1.39814400
O	-2.90629999	-0.10527998	4.32523645
O	-2.81610824	0.01302928	-4.25898857

[1,5,9] - $[Mo_3V_7O_{28}]^{3-}$ Symmol = NOSYM

Mo	0.00447162	2.36137049	1.57219891
V	0.04123132	2.28768545	-1.58623923
V	0.03277078	-2.30702904	1.50651239
V	0.03002079	-2.28746546	-1.54861180
Mo	-1.60903156	-0.07229691	3.17243007
V	1.63241957	-0.06702908	3.08978507
V	-1.64630699	-0.01337975	-0.00636778
V	1.62396162	-0.00568889	-0.00920239
Mo	-1.60001246	-0.00588613	-3.17981563
V	1.63160228	-0.01582738	-3.11478123
O	2.62073203	0.01098369	1.39478260
O	2.63136279	0.00142000	-1.38416794
O	1.24550358	-1.89128254	0.01403504
O	1.25189306	1.92539948	-0.01070175
O	-0.02706798	0.03260203	1.33695869
O	-0.03081774	0.00722369	-1.27919213
O	1.32072458	-1.84131460	2.73090240
O	1.35442999	1.84716977	2.74222027
O	2.79376096	-0.05632045	4.20115786
O	0.03073638	0.01944145	4.10100204
O	1.32940541	-1.82429813	-2.71512776
O	1.33645740	1.80444304	-2.74307579
O	0.03497346	-0.01709548	-4.12227871
O	2.80222883	-0.02716323	-4.21770557
O	-1.24035519	-1.87764498	0.02394440
O	0.02374545	-3.91681096	1.52181590
O	-1.34293411	-1.88829748	2.74500698
O	0.01738913	-3.90168634	-1.54380639
O	-1.35324091	-1.85477887	-2.71533439
O	-1.24685798	1.91726417	-0.00432952
O	-1.34923537	1.84694085	-2.74386638
O	0.03463574	3.90346039	-1.59635427
O	-1.39218633	1.88019100	2.76023076
O	0.01050615	4.08333123	1.57682464
O	-2.67279540	0.00651880	-1.38402312
O	-2.65668497	0.00986481	1.41088711
O	-2.83173331	-0.06144050	4.37584913
O	-2.83570261	-0.01257412	-4.37156251

[1,5,10] - $[Mo_3V_7O_{28}]^{3-}$ Symmol = NOSYM

Mo	0.05133903	2.35662286	1.57579948
V	-0.06111112	2.29034904	-1.58527415
V	0.07855600	-2.30940904	1.50844863
V	-0.08699496	-2.28323317	-1.55294625
Mo	-1.60189945	-0.07104925	3.14589734
V	1.63869753	-0.07021977	3.12040019
V	-1.63580881	-0.00438323	-0.01543794
V	1.64178422	-0.01429399	-0.00253482
V	-1.63283060	0.00232835	-3.14630456
Mo	1.60101652	-0.02091617	-3.15708563
O	2.60781251	0.01637370	1.43540243
O	2.68967830	0.00529900	-1.35531256
O	1.25913736	-1.88845484	0.04152850
O	1.25223826	1.92230484	0.00818227
O	-0.03585376	0.03810201	1.33994805
O	0.04298568	-0.00469148	-1.27000590
O	1.32393088	-1.84529917	2.76593519
O	1.36166754	1.85601694	2.77995896
O	2.78974683	-0.05862853	4.24145953
O	0.02160517	0.02262719	4.10484667
O	1.34371704	-1.85618236	-2.69390659
O	1.34605880	1.83379066	-2.72086424
O	-0.02676042	-0.01675425	-4.12334463
O	2.85203767	-0.03039199	-4.33378360
O	-1.23432360	-1.88629610	0.00714558
O	0.05758782	-3.91903740	1.52609670
O	-1.33583102	-1.88310949	2.71847616
O	-0.06043380	-3.89793680	-1.54863793
O	-1.34224037	-1.82147456	-2.74425231
O	-1.24763452	1.91771134	-0.03034464
O	-1.33451033	1.81437522	-2.76591073
O	-0.03605512	3.90534239	-1.59867018
O	-1.38927655	1.87462620	2.72476136
O	0.02481472	4.07867695	1.57460184
O	-2.61567743	0.00206741	-1.41721115
O	-2.66805146	0.00496635	1.37189509
O	-2.84563511	-0.05837972	4.32944587
O	-2.79348345	-0.00143918	-4.25840203

[1,6,7] - $[Mo_3V_7O_{28}]^{3-}$ Symmol = NOSYM

Mo	0.03049615	2.35294379	1.60712264
V	-0.00721162	2.26776205	-1.65267182
V	-0.08173910	-2.30393211	1.53011325
V	0.03859368	-2.26630770	-1.61460669
V	-1.57260787	-0.02890125	3.19181650
Mo	1.65657209	-0.11216511	3.15266880
Mo	-1.75700675	0.01926855	-0.03717959
V	1.61651610	-0.02554799	-0.01010860
V	-1.51937815	-0.03270084	-3.26643325
V	1.62723668	0.00997450	-3.17418334
O	2.65576477	0.02635208	1.39821563
O	2.59703490	0.01901861	-1.38674932
O	1.20113803	-1.86805776	0.02942823
O	1.19245550	1.91468365	-0.00336197
O	0.00533212	0.03895221	1.38858449
O	-0.05764097	0.00473934	-1.22703112
O	1.35194743	-1.90855239	2.73994770
O	1.40926454	1.88688214	2.77877644
O	2.90636258	-0.10576811	4.32529989
O	0.03963481	0.01964234	4.14212971
O	1.36269479	-1.80408164	-2.72956714
O	1.33559017	1.81684829	-2.75609376
O	0.06326922	-0.01826255	-4.14847914
O	2.81616695	0.01301815	-4.25893299
O	-1.29509187	-1.90390085	0.03770918
O	-0.08231512	-3.91206799	1.54021905
O	-1.30924860	-1.82329579	2.78416683
O	0.02481848	-3.88308568	-1.58795514
O	-1.31416923	-1.80523092	-2.74022271
O	-1.31433866	1.93294801	0.01252476
O	-1.34063659	1.76385066	-2.76977384
O	-0.05201606	3.88517949	-1.64384580
O	-1.34455202	1.84546733	2.78077524
O	-0.01835847	4.07233446	1.58452184
O	-2.80317685	-0.01208505	-1.46138387
O	-2.69412374	-0.00125040	1.51239770
O	-2.70189582	-0.02067585	4.33390660
O	-2.66538149	-0.05399566	-4.40174440

[1,6,8] - $[Mo_3V_7O_{28}]^{3-}$ Symmol = NOSYM

Mo	-0.05981020	2.34851632	1.60060555
V	0.01946067	2.26498444	-1.65181697
V	-0.04434370	-2.30012890	1.53749523
V	0.00511057	-2.26258524	-1.61761306
V	-1.68891899	-0.08922585	3.09486093
Mo	1.54069719	-0.04794159	3.25409644
V	-1.61426290	-0.01608421	-0.03178688
Mo	1.76786614	-0.00015010	-0.02042977
V	-1.61928497	-0.00387576	-3.15864522
V	1.53091985	-0.01694108	-3.28559963
O	2.76676233	0.00608511	1.49183759
O	2.74817291	-0.00028411	-1.50243259
O	1.28664701	-1.89320166	0.02272387
O	1.30186766	1.92414818	-0.00858654
O	0.08661014	0.03952850	1.39951670
O	0.02640467	0.00291962	-1.22009961
O	1.33556667	-1.86341110	2.76986174
O	1.38042288	1.87282645	2.77581781
O	2.76354879	-0.03601851	4.46022029
O	-0.08953724	0.01896288	4.15716265
O	1.32441868	-1.79885424	-2.76507414
O	1.32971259	1.77945326	-2.79256282
O	-0.06556687	-0.01658365	-4.14682317
O	2.65979598	-0.02770043	-4.43606578
O	-1.21031017	-1.87829622	0.04426961
O	-0.00464895	-3.90769402	1.54224030
O	-1.33045721	-1.85648818	2.76186319
O	0.02004212	-3.87925323	-1.58802144
O	-1.35092076	-1.80879539	-2.70997621
O	-1.21013717	1.91271166	0.01434255
O	-1.34526217	1.80182182	-2.73245668
O	0.03770204	3.88287044	-1.63700744
O	-1.37347154	1.85502641	2.78928507
O	-0.00913434	4.06824543	1.58953179
O	-2.63993462	0.00470195	-1.36278018
O	-2.59519694	0.01626057	1.41336603
O	-2.86551523	-0.08721602	4.18568876
O	-2.81501493	-0.00833356	-4.23700799

[1,6,9] - $[Mo_3V_7O_{28}]^{3-}$ Symmol = NOSYM

Mo	-0.05140549	2.35662009	1.57578315
V	0.06119235	2.29035408	-1.58526139
V	-0.07847818	-2.30940554	1.50844320
V	0.08693488	-2.28323495	-1.55294964
V	-1.63869727	-0.07027499	3.12038518
Mo	1.60188739	-0.07099797	3.14590863
V	-1.64179237	-0.01433389	-0.00254566
V	1.63584021	-0.00437002	-0.01544043
Mo	-1.60102207	-0.02083249	-3.15707642
V	1.63281684	0.00225022	-3.14630780
O	2.66807752	0.00501841	1.37189988
O	2.61569014	0.00205072	-1.41722570
O	1.23433121	-1.88626614	0.00714064
O	1.24766370	1.91769905	-0.03033536
O	0.03582188	0.03808989	1.33996126
O	-0.04299193	-0.00470034	-1.26999127
O	1.33587300	-1.88308203	2.71847332
O	1.38918824	1.87461404	2.72479535
O	2.84562795	-0.05830401	4.32945207
O	-0.02161688	0.02263338	4.10486072
O	1.34222567	-1.82150346	-2.74425557
O	1.33452604	1.81433575	-2.76592213
O	0.02673048	-0.01673543	-4.12336534
O	2.79345646	-0.00153738	-4.25841643
O	-1.25913567	-1.88850189	0.04153492
O	-0.05749147	-3.91903262	1.52611070
O	-1.32390575	-1.84531913	2.76592619
O	0.06036348	-3.89793778	-1.54862606
O	-1.34369698	-1.85613610	-2.69391806
O	-1.25221397	1.92231824	0.00815193
O	-1.34604607	1.83382832	-2.72082414
O	0.03616447	3.90534716	-1.59867293
O	-1.36170582	1.85599699	2.77993189
O	-0.02485711	4.07867516	1.57460477
O	-2.68970190	0.00530177	-1.35530791
O	-2.60782968	0.01636070	1.43537995
O	-2.78975127	-0.05868608	4.24144193
O	-2.85207203	-0.03030173	-4.33374345

[1,6,10] - $[Mo_3V_7O_{28}]^{3-}$ Symmol = NOSYM

Mo	-0.00041771	2.35932901	1.57496814
V	-0.04504656	2.28750446	-1.58311830
V	-0.03488557	-2.30667526	1.50684114
V	-0.02975951	-2.28644295	-1.55045917
V	-1.63142926	-0.06489556	3.08876151
Mo	1.60945398	-0.07530551	3.17096288
V	-1.62645171	-0.00563026	-0.01183320
V	1.64689341	-0.01558529	-0.00646773
V	-1.63120636	-0.01701530	-3.11696118
Mo	1.60025360	-0.00266217	-3.17923718
O	2.65656604	0.01209027	1.41154580
O	2.67235680	0.00685656	-1.38456243
O	1.24106695	-1.87836962	0.02651472
O	1.24617752	1.91548240	-0.00555793
O	0.02950400	0.03371874	1.34040750
O	0.03094728	0.00462066	-1.27585959
O	1.34261063	-1.89049282	2.74624363
O	1.39468181	1.88141345	2.76204888
O	2.83195621	-0.06374031	4.37463671
O	-0.02894165	0.02130138	4.10058159
O	1.35388688	-1.85304519	-2.71509994
O	1.34657034	1.84733559	-2.74196373
O	-0.03403399	-0.01796877	-4.12200619
O	2.83612215	-0.00856025	-4.37057619
O	-1.24602453	-1.89129506	0.01464480
O	-0.02683368	-3.91647791	1.52210669
O	-1.32025367	-1.84005660	2.73210792
O	-0.01622583	-3.90072014	-1.54459229
O	-1.32990398	-1.82470126	-2.71559863
O	-1.25201512	1.92328789	-0.01106339
O	-1.33637685	1.80280883	-2.74344144
O	-0.03850295	3.90318955	-1.59487612
O	-1.35532632	1.84931265	2.74079653
O	-0.00826880	4.08122756	1.57248034
O	-2.63362755	0.00129590	-1.38643549
O	-2.62022772	0.01060135	1.39450979
O	-2.79250628	-0.05327849	4.20034918
O	-2.80078201	-0.02845753	-4.22079764

[1,7,8] - $[Mo_3V_7O_{28}]^{3-}$ Symmol = C(S)

Mo	1.63622259	2.33423607	0.00000000
V	-1.66466358	2.25948789	0.00000000
V	1.62847306	-2.28627710	0.00000000
V	-1.65559844	-2.26344575	0.00000000
V	3.23650896	-0.05668130	1.57892296
V	3.23650896	-0.05668130	-1.57892296
Mo	-0.00489019	-0.02974847	1.73104610
Mo	-0.00489019	-0.02974847	-1.73104610
V	-3.25581217	-0.00090582	1.57314512
V	-3.25581217	-0.00090582	-1.57314512
O	1.50096053	0.00552052	-2.70584817
O	-1.47985029	0.00328488	-2.72916865
O	0.02199431	-1.90413214	-1.24702612
O	-0.00238889	1.91416842	-1.25733063
O	1.33489042	0.02467768	0.00000000
O	-1.27537931	0.00086947	0.00000000
O	2.78743298	-1.82811279	-1.33218795
O	2.78513694	1.83456703	-1.37043560
O	4.36158289	-0.04905277	-2.72643273
O	4.15298193	0.01891926	0.00000000
O	-2.76755637	-1.80035033	-1.33939087
O	-2.78090984	1.79586357	-1.33909196
O	-4.18198644	-0.00655526	0.00000000
O	-4.37298485	-0.00290825	-2.73260620
O	0.02199431	-1.90413214	1.24702612
O	1.61339583	-3.89700911	0.00000000
O	2.78743298	-1.82811279	1.33218795
O	-1.62387529	-3.87959157	0.00000000
O	-2.76755637	-1.80035033	1.33939087
O	-0.00238889	1.91416842	1.25733063
O	-2.78090984	1.79586357	1.33909196
O	-1.63513630	3.87669240	0.00000000
O	2.78513694	1.83456703	1.37043560
O	1.61222750	4.05497089	0.00000000
O	-1.47985029	0.00328488	2.72916865
O	1.50096053	0.00552052	2.70584817
O	4.36158289	-0.04905277	2.72643273
O	-4.37298485	-0.00290825	2.73260620

[1,7,9] - $[Mo_3V_7O_{28}]^{3-}$ Symmol = NOSYM

Mo	0.00097133	2.33678475	1.61768877
V	0.04399131	2.27930086	-1.60529549
V	-0.01939677	-2.28940660	1.59451895
V	0.07069617	-2.27688351	-1.58083370
V	-1.53280345	-0.04806796	3.25176466
V	1.62846826	-0.07803157	3.13433228
Mo	-1.76966878	-0.02281129	0.01234355
V	1.60956420	-0.01904170	0.00976306
Mo	-1.54083775	-0.01455783	-3.25873709
V	1.68177118	0.00546732	-3.11423171
O	2.61189807	0.00395160	1.38209992
O	2.60833014	0.00521025	-1.39210019
O	1.19160396	-1.87951624	0.00086567
O	1.20482547	1.90615689	-0.02892905
O	-0.04229071	0.03081873	1.29219150
O	-0.08572012	0.00083299	-1.32860221
O	1.34120919	-1.84695099	2.73033802
O	1.38553704	1.83749237	2.73622667
O	2.81251746	-0.07326974	4.22219207
O	0.06671751	0.01501426	4.11029673
O	1.35154160	-1.82031151	-2.74489404
O	1.34106550	1.82041770	-2.76173855
O	0.09245093	-0.01078619	-4.16943752
O	2.86729364	0.00491284	-4.19733883
O	-1.29893014	-1.90072040	0.02702602
O	-0.04085961	-3.90144109	1.58118346
O	-1.32118824	-1.82085793	2.78708178
O	0.02951385	-3.89020009	-1.56994341
O	-1.33777107	-1.84653608	-2.74609218
O	-1.30320044	1.92044031	-0.00274105
O	-1.34564649	1.83187160	-2.77038459
O	0.00249049	3.89343344	-1.60117954
O	-1.35759657	1.83477219	2.78934887
O	-0.01608949	4.05936909	1.59879816
O	-2.78617567	0.00410440	-1.47663490
O	-2.72592096	0.01108831	1.52527676
O	-2.65195840	-0.03912767	4.40858446
O	-2.76640264	-0.02292151	-4.46280734

[1,7,10] - $[Mo_3V_7O_{28}]^{3-}$ Symmol = NOSYM

Mo	0.04561587	2.33798725	1.61732693
V	-0.05987095	2.28431812	-1.60442270
V	0.01536765	-2.29214531	1.59413202
V	-0.02725345	-2.28126689	-1.57877874
V	-1.52873271	-0.04332365	3.22312106
V	1.63104601	-0.08256093	3.16492446
Mo	-1.74651009	-0.01748096	-0.00624935
V	1.61872507	-0.02739170	0.02361112
V	-1.57642454	-0.01823540	-3.21650711
Mo	1.64954872	0.00886080	-3.15905235
O	2.60036545	0.00468933	1.41769841
O	2.65231727	0.00659701	-1.36541144
O	1.19511672	-1.87642386	0.01902387
O	1.20408982	1.90656496	-0.01067338
O	-0.04120596	0.03260949	1.29326936
O	-0.02052755	0.00265404	-1.31853234
O	1.34645914	-1.85370656	2.75736919
O	1.39468252	1.84321777	2.77070086
O	2.80269248	-0.08010988	4.26555739
O	0.05790049	0.01383136	4.11361040
O	1.36165090	-1.85054194	-2.72145268
O	1.35195639	1.85592960	-2.74018582
O	0.03020004	-0.01155927	-4.15767911
O	2.90989979	0.00702749	-4.32115511
O	-1.28930783	-1.90974017	0.00024528
O	-0.01762784	-3.90410737	1.58441749
O	-1.31634589	-1.81445334	2.75905766
O	-0.03460215	-3.89431852	-1.56602713
O	-1.32194800	-1.81595140	-2.76102458
O	-1.30121619	1.93102623	-0.02745410
O	-1.33061030	1.79987290	-2.78782805
O	-0.06734535	3.89858048	-1.60269366
O	-1.34952097	1.82828738	2.75768050
O	0.00711320	4.06039841	1.60251178
O	-2.73418041	0.00308936	-1.50587885
O	-2.73338471	0.00808849	1.48000762
O	-2.66684481	-0.03261796	4.36250883
O	-2.71128785	-0.02769540	-4.35576777

[1,8,9] - $[Mo_3V_7O_{28}]^{3-}$ Symmol = NOSYM

Mo	-0.04557452	2.33799700	1.61731261
V	0.05979142	2.28433155	-1.60441835
V	-0.01543684	-2.29214444	1.59414789
V	0.02736699	-2.28128657	-1.57883339
V	-1.63105746	-0.08250796	3.16491332
V	1.52873589	-0.04335588	3.22312628
V	-1.61869867	-0.02742907	0.02361493
Mo	1.74652564	-0.01748862	-0.00624607
Mo	-1.64955937	0.00878869	-3.15895249
V	1.57642184	-0.01815432	-3.21652978
O	2.73339217	0.00806185	1.48001859
O	2.73416708	0.00303813	-1.50590169
O	1.28931056	-1.90972306	0.00023486
O	1.30122611	1.93096814	-0.02746038
O	0.04118412	0.03259222	1.29325799
O	0.02049612	0.00261978	-1.31856582
O	1.31630526	-1.81445999	2.75907542
O	1.34952265	1.82826580	2.75769423
O	2.66683165	-0.03265893	4.36252930
O	-0.05792368	0.01386783	4.11359469
O	1.32195538	-1.81598082	-2.76112293
O	1.33062931	1.79988027	-2.78777897
O	-0.03021635	-0.01147533	-4.15759971
O	2.71126144	-0.02757337	-4.35581541
O	-1.19511195	-1.87645364	0.01902008
O	0.01757423	-3.90410588	1.58443224
O	-1.34650103	-1.85370609	2.75739127
O	0.03471199	-3.89433826	-1.56604236
O	-1.36161277	-1.85060199	-2.72148894
O	-1.20405734	1.90658596	-0.01069357
O	-1.35194876	1.85594230	-2.74018667
O	0.06732341	3.89859322	-1.60267557
O	-1.39468070	1.84326567	2.77065078
O	-0.00704547	4.06040681	1.60249788
O	-2.65236993	0.00658216	-1.36536664
O	-2.60033347	0.00471937	1.41770305
O	-2.80271070	-0.08001762	4.26553776
O	-2.90989425	0.00695509	-4.32107443

[1,8,10] - $[Mo_3V_7O_{28}]^{3-}$ Symmol = NOSYM

Mo	-0.00098270	2.33679293	1.61769573
V	-0.04396910	2.27931689	-1.60532577
V	0.01941300	-2.28942197	1.59452376
V	-0.07074759	-2.27689297	-1.58082014
V	-1.62847099	-0.07804561	3.13429259
V	1.53279378	-0.04807549	3.25178447
V	-1.60953403	-0.01902674	0.00976776
Mo	1.76967986	-0.02278844	0.01235614
V	-1.68174528	0.00549475	-3.11423458
Mo	1.54085888	-0.01460241	-3.25869793
O	2.72593182	0.01110667	1.52528452
O	2.78616584	0.00412928	-1.47665171
O	1.29893501	-1.90066325	0.02701647
O	1.30321138	1.92041926	-0.00276762
O	0.04227218	0.03083013	1.29221309
O	0.08570064	0.00084710	-1.32862999
O	1.32120954	-1.82085515	2.78708216
O	1.35757403	1.83476471	2.78936747
O	2.65195329	-0.03912867	4.40859949
O	-0.06673545	0.01501026	4.11029988
O	1.33774242	-1.84655717	-2.74607716
O	1.34559459	1.83189695	-2.77049518
O	-0.09245054	-0.01081082	-4.16941602
O	2.76640669	-0.02297264	-4.46278483
O	-1.19158603	-1.87954016	0.00089561
O	0.04091827	-3.90145529	1.58116587
O	-1.34116942	-1.84699461	2.73036078
O	-0.02955259	-3.89020924	-1.56994025
O	-1.35158755	-1.82032150	-2.74486032
O	-1.20479476	1.90618488	-0.02891665
O	-1.34107945	1.82044648	-2.76173278
O	-0.00247572	3.89344978	-1.60118252
O	-1.38556427	1.83750394	2.73620281
O	0.01610602	4.05937751	1.59881613
O	-2.60829807	0.00524538	-1.39207667
O	-2.61189709	0.00392056	1.38209495
O	-2.81254652	-0.07332218	4.22211913
O	-2.86728010	0.00494686	-4.19732870

[1,9,10] - $[Mo_3V_7O_{28}]^{3-}$ Symmol = C(S)

Mo	0.00000000	2.34405093	1.59699947
V	0.00000000	2.29988249	-1.54781593
V	0.00000000	-2.29428296	1.56575961
V	0.00000000	-2.29481475	-1.50218080
V	-1.58351777	-0.06635796	3.15692629
V	1.58351777	-0.06635796	3.15692629
V	-1.63890989	-0.01511459	0.03251678
V	1.63890989	-0.01511459	0.03251678
Mo	-1.64852606	-0.00966718	-3.13087125
Mo	1.64852606	-0.00966718	-3.13087125
O	2.62870797	0.00655079	1.40409843
O	2.66915999	0.00823323	-1.38567354
O	1.24143659	-1.88210984	-0.00101182
O	1.24941302	1.91634665	-0.02810058
O	0.00000000	0.03230869	1.24651632
O	0.00000000	0.00523781	-1.37929587
O	1.33076399	-1.83709681	2.73265443
O	1.37038348	1.83394982	2.74146334
O	2.73975231	-0.06000903	4.27691328
O	0.00000000	0.01082984	4.07444412
O	1.34315476	-1.86414062	-2.72623191
O	1.34364379	1.85687968	-2.75418158
O	0.00000000	-0.01292230	-4.15436701
O	2.89136676	-0.01611420	-4.31372947
O	-1.24143659	-1.88210984	-0.00101182
O	0.00000000	-3.90741476	1.56048509
O	-1.33076399	-1.83709681	2.73265443
O	0.00000000	-3.90565894	-1.51009383
O	-1.34315476	-1.86414062	-2.72623191
O	-1.24941302	1.91634665	-0.02810058
O	-1.34364379	1.85687968	-2.75418158
O	0.00000000	3.91248324	-1.55936061
O	-1.37038348	1.83394982	2.74146334
O	0.00000000	4.06760083	1.59936465
O	-2.66915999	0.00823323	-1.38567354
O	-2.62870797	0.00655079	1.40409843
O	-2.73975231	-0.06000903	4.27691328
O	-2.89136676	-0.01611420	-4.31372947

[5,6,7] - $[Mo_3V_7O_{28}]^{3-}$ Symmol = C(S)

V	0.01449952	1.53121174	-2.28901852
V	0.00763283	-1.62138752	-2.26339723
V	0.01449952	1.53121174	2.28901852
V	0.00763283	-1.62138752	2.26339723
Mo	-1.58393896	3.23183727	0.00000000
Mo	1.69863736	3.12760059	0.00000000
Mo	-1.75966525	-0.02785816	0.00000000
V	1.61519761	-0.04055164	0.00000000
V	-1.52749614	-3.27160814	0.00000000
V	1.61676357	-3.17959516	0.00000000
O	2.65771162	1.38985961	0.00000000
O	2.62424976	-1.38168180	0.00000000
O	1.20260043	0.02808402	1.87697001
O	1.20260043	0.02808402	-1.87697001
O	-0.04692384	1.41356246	0.00000000
O	-0.03539947	-1.22289617	0.00000000
O	1.34367683	2.75708122	1.85825102
O	1.34367683	2.75708122	-1.85825102
O	2.95424147	4.29215570	0.00000000
O	0.05811451	4.20787418	0.00000000
O	1.34878856	-2.73059771	1.80407086
O	1.34878856	-2.73059771	-1.80407086
O	0.05982895	-4.15383519	0.00000000
O	2.80710836	-4.26361459	0.00000000
O	-1.29274005	0.02038018	1.90144486
O	-0.01466053	1.54072318	3.89881112
O	-1.33564070	2.76617569	1.84397068
O	-0.01455552	-1.60411475	3.88045003
O	-1.32146281	-2.76006240	1.78412927
O	-1.29274005	0.02038018	-1.90144486
O	-1.32146281	-2.76006240	-1.78412927
O	-0.01455552	-1.60411475	-3.88045003
O	-1.33564070	2.76617569	-1.84397068
O	-0.01466053	1.54072318	-3.89881112
O	-2.76639909	-1.48416007	0.00000000
O	-2.76494112	1.49057324	0.00000000
O	-2.81455203	4.42808314	0.00000000
O	-2.66881438	-4.41073257	0.00000000

[5,6,9] - $[Mo_3V_7O_{28}]^{3-}$ Symmol = C(S)

V	-0.03134782	2.29433793	1.50178393
V	0.06226040	2.28429360	-1.55317146
V	-0.03134782	-2.29433793	1.50178393
V	0.06226040	-2.28429360	-1.55317146
Mo	-1.64819626	0.00000000	3.15801636
Mo	1.64830309	0.00000000	3.12217495
V	-1.64239667	0.00000000	-0.00197286
V	1.62933567	0.00000000	-0.03650538
Mo	-1.60209745	0.00000000	-3.17071059
V	1.62660344	0.00000000	-3.14119796
O	2.66577483	0.00000000	1.36671932
O	2.63407613	0.00000000	-1.40700746
O	1.24283018	-1.89115068	0.00148659
O	1.24283018	1.89115068	0.00148659
O	-0.01262777	0.00000000	1.36069612
O	-0.02358294	0.00000000	-1.26858045
O	1.33212792	-1.84751553	2.71922211
O	1.33212792	1.84751553	2.71922211
O	2.90005719	0.00000000	4.29592656
O	0.00405409	0.00000000	4.16154532
O	1.33651740	-1.81327656	-2.73834287
O	1.33651740	1.81327656	-2.73834287
O	0.03071671	0.00000000	-4.12871231
O	2.79209072	0.00000000	-4.24889332
O	-1.24651335	-1.88578357	0.02166030
O	-0.01703921	-3.90604384	1.52349354
O	-1.34446409	-1.85683661	2.75225922
O	0.04052449	-3.89887796	-1.56139634
O	-1.34020782	-1.84169578	-2.71466597
O	-1.24651335	1.88578357	0.02166030
O	-1.34020782	1.84169578	-2.71466597
O	0.04052449	3.89887796	-1.56139634
O	-1.34446409	1.85683661	2.75225922
O	-0.01703921	3.90604384	1.52349354
O	-2.67118830	0.00000000	-1.37204668
O	-2.67251112	0.00000000	1.41146768
O	-2.88238826	0.00000000	4.34903353
O	-2.84539931	0.00000000	-4.35461090

[5,7,8] - $[Mo_3V_7O_{28}]^{3-}$ Symmol = C(S)

V	0.05222247	1.60074420	-2.27439270
V	-0.02239323	-1.64478781	-2.26197341
V	0.05222247	1.60074420	2.27439270
V	-0.02239323	-1.64478781	2.26197341
Mo	-1.58533171	3.25666140	0.00000000
V	1.62766131	3.21177068	0.00000000
Mo	-1.74006860	-0.01017015	0.00000000
Mo	1.72336809	-0.01955909	0.00000000
V	-1.57884331	-3.27558728	0.00000000
V	1.56627889	-3.24737998	0.00000000
O	2.69313832	1.50240144	0.00000000
O	2.75551616	-1.46070151	0.00000000
O	1.25084157	0.02953145	1.90190244
O	1.25084157	0.02953145	-1.90190244
O	-0.04926637	1.36337930	0.00000000
O	0.01433682	-1.25856602	0.00000000
O	1.33055519	2.78516703	1.80764435
O	1.33055519	2.78516703	-1.80764435
O	2.78389997	4.32488856	0.00000000
O	0.03484159	4.21197810	0.00000000
O	1.33266873	-2.75157771	1.78883952
O	1.33266873	-2.75157771	-1.78883952
O	0.00169098	-4.18599251	0.00000000
O	2.73410472	-4.35686062	0.00000000
O	-1.24885022	-0.00558723	1.89249453
O	0.03009036	1.59424211	3.88637067
O	-1.34845823	2.76479278	1.83855542
O	-0.01328263	-1.62131383	3.87829420
O	-1.33762266	-2.78476149	1.79570029
O	-1.24885022	-0.00558723	-1.89249453
O	-1.33762266	-2.78476149	-1.79570029
O	-0.01328263	-1.62131383	-3.87829420
O	-1.34845823	2.76479278	-1.83855542
O	0.03009036	1.59424211	-3.88637067
O	-2.71019703	-1.50612670	0.00000000
O	-2.76843471	1.47615329	0.00000000
O	-2.82499243	4.44206910	0.00000000
O	-2.72924539	-4.40125701	0.00000000

[5,7,9] - $[Mo_3V_7O_{28}]^{3-}$ Symmol = C(2V)

V	1.57362407	2.27665877	-0.04168270
V	-1.57362407	2.27665877	-0.04168270
V	1.57362407	-2.27665877	-0.04168270
V	-1.57362407	-2.27665877	-0.04168270
Mo	3.27257098	0.00000000	1.54595254
V	3.11088611	0.00000000	-1.67321211
Mo	0.00000000	-0.00000000	1.77693024
V	0.00000000	-0.00000000	-1.60080052
Mo	-3.27257098	-0.00000000	1.54595254
V	-3.11088611	-0.00000000	-1.67321211
O	1.37857138	0.00000000	-2.61321773
O	-1.37857138	-0.00000000	-2.61321773
O	0.00000000	-1.88585605	-1.19585907
O	-0.00000000	1.88585605	-1.19585907
O	1.31582890	0.00000000	0.07609840
O	-1.31582890	-0.00000000	0.07609840
O	2.73194682	-1.81412658	-1.34139087
O	2.73194682	1.81412658	-1.34139087
O	4.18722344	0.00000000	-2.86576729
O	4.17087536	0.00000000	-0.09783299
O	-2.73194682	-1.81412658	-1.34139087
O	-2.73194682	1.81412658	-1.34139087
O	-4.17087536	-0.00000000	-0.09783299
O	-4.18722344	-0.00000000	-2.86576729
O	0.00000000	-1.89393301	1.29917460
O	1.57303661	-3.89032089	-0.00422207
O	2.76687813	-1.83475529	1.33759373
O	-1.57303661	-3.89032089	-0.00422207
O	-2.76687813	-1.83475529	1.33759373
O	-0.00000000	1.89393301	1.29917460
O	-2.76687813	1.83475529	1.33759373
O	-1.57303661	3.89032089	-0.00422207
O	2.76687813	1.83475529	1.33759373
O	1.57303661	3.89032089	-0.00422207
O	-1.50107739	-0.00000000	2.77902776
O	1.50107739	0.00000000	2.77902776
O	4.49013973	0.00000000	2.75697484
O	-4.49013973	-0.00000000	2.75697484

[5,7,10] - $[Mo_3V_7O_{28}]^{3-}$ Symmol = C(S)

V	0.08433946	1.57704307	-2.27615128
V	-0.06334787	-1.57606836	-2.27911520
V	0.08433946	1.57704307	2.27615128
V	-0.06334787	-1.57606836	2.27911520
Mo	-1.53804168	3.24993057	0.00000000
V	1.67999607	3.14027004	0.00000000
Mo	-1.75880253	-0.00608500	0.00000000
V	1.61620748	0.00002472	0.00000000
V	-1.58429659	-3.23435706	0.00000000
Mo	1.64058839	-3.15594801	0.00000000
O	2.59603268	1.41426218	0.00000000
O	2.67325604	-1.35328727	0.00000000
O	1.19845505	0.02167473	1.88006473
O	1.19845505	0.02167473	-1.88006473
O	-0.09084052	1.31966742	0.00000000
O	0.00187054	-1.30336319	0.00000000
O	1.34630463	2.76662122	1.81815588
O	1.34630463	2.76662122	-1.81815588
O	2.86269706	4.22621777	0.00000000
O	0.08724582	4.17895046	0.00000000
O	1.35431680	-2.71165194	1.84524918
O	1.35431680	-2.71165194	-1.84524918
O	0.03099941	-4.16392101	0.00000000
O	2.90902415	-4.31045635	0.00000000
O	-1.29791665	-0.02988718	1.90410061
O	0.03215221	1.57777477	3.88975000
O	-1.33490749	2.73996864	1.83017103
O	-0.06071974	-1.57477843	3.89265761
O	-1.32049475	-2.78596577	1.80362972
O	-1.29791665	-0.02988718	-1.90410061
O	-1.32049475	-2.78596577	-1.80362972
O	-0.06071974	-1.57477843	-3.89265761
O	-1.33490749	2.73996864	-1.83017103
O	0.03215221	1.57777477	-3.88975000
O	-2.71430166	-1.53048368	0.00000000
O	-2.79883702	1.45662112	0.00000000
O	-2.77675673	4.44176322	0.00000000
O	-2.71240425	-4.37926741	0.00000000

[5,8,9] - $[Mo_3V_7O_{28}]^{3-}$ Symmol = C(2V)

V	1.57182760	2.28240522	-0.01839101
V	-1.57182760	2.28240522	-0.01839101
V	1.57182760	-2.28240522	-0.01839101
V	-1.57182760	-2.28240522	-0.01839101
Mo	3.18300586	0.00000000	1.64685009
V	3.20246774	0.00000000	-1.57608508
V	0.00000000	0.00000000	1.62083758
Mo	0.00000000	0.00000000	-1.73468734
Mo	-3.18300586	-0.00000000	1.64685009
V	-3.20246774	-0.00000000	-1.57608508
O	1.48471154	0.00000000	-2.73476766
O	-1.48471154	-0.00000000	-2.73476766
O	0.00000000	-1.91546304	-1.29188921
O	-0.00000000	1.91546304	-1.29188921
O	1.30892376	0.00000000	-0.01408230
O	-1.30892376	-0.00000000	-0.01408230
O	2.75234009	-1.79810883	-1.31623022
O	2.75234009	1.79810883	-1.31623022
O	4.33164892	0.00000000	-2.72224412
O	4.16370218	0.00000000	0.02340839
O	-2.75234009	-1.79810883	-1.31623022
O	-2.75234009	1.79810883	-1.31623022
O	-4.16370218	-0.00000000	0.02340839
O	-4.33164892	-0.00000000	-2.72224412
O	0.00000000	-1.87789472	1.19489803
O	1.57454922	-3.89599104	-0.03449154
O	2.74001334	-1.85039677	1.35958302
O	-1.57454922	-3.89599104	-0.03449154
O	-2.74001334	-1.85039677	1.35958302
O	-0.00000000	1.87789472	1.19489803
O	-2.74001334	1.85039677	1.35958302
O	-1.57454922	3.89599104	-0.03449154
O	2.74001334	1.85039677	1.35958302
O	1.57454922	3.89599104	-0.03449154
O	-1.38750847	-0.00000000	2.65093448
O	1.38750847	0.00000000	2.65093448
O	4.35357825	0.00000000	2.89896179
O	-4.35357825	-0.00000000	2.89896179

[1,4] - $[Mo_2V_8O_{28}]^{6-}$ (spin polarised) Symmol = C(2H)

Mo	1.60168828	2.35345826	0.00000000
V	-1.61835137	2.31263730	0.00000000
V	1.61835137	-2.31263730	0.00000000
Mo	-1.60168828	-2.35345826	0.00000000
V	3.11385787	-0.06015153	1.59699837
V	3.11385787	-0.06015153	-1.59699837
V	0.00000000	0.00000000	1.62157382
V	-0.00000000	-0.00000000	-1.62157382
V	-3.11385787	0.06015153	1.59699837
V	-3.11385787	0.06015153	-1.59699837
O	1.40354113	-0.00201161	-2.63318963
O	-1.40354113	0.00201161	-2.63318963
O	0.01716869	-1.92734673	-1.24021248
O	-0.01716869	1.92734673	-1.24021248
O	1.31873427	0.02947646	0.00000000
O	-1.31873427	-0.02947646	0.00000000
O	2.74788843	-1.87965121	-1.35542596
O	2.74174265	1.88896507	-1.38684237
O	4.24339011	-0.03737402	-2.77783838
O	4.06089344	0.02270312	0.00000000
O	-2.74174265	-1.88896507	-1.38684237
O	-2.74788843	1.87965121	-1.35542596
O	-4.06089344	-0.02270312	0.00000000
O	-4.24339011	0.03737402	-2.77783838
O	0.01716869	-1.92734673	1.24021248
O	1.59461448	-3.94938583	0.00000000
O	2.74788843	-1.87965121	1.35542596
O	-1.57198516	-4.09903754	0.00000000
O	-2.74174265	-1.88896507	1.38684237
O	-0.01716869	1.92734673	1.24021248
O	-2.74788843	1.87965121	1.35542596
O	-1.59461448	3.94938583	0.00000000
O	2.74174265	1.88896507	1.38684237
O	1.57198516	4.09903754	0.00000000
O	-1.40354113	0.00201161	2.63318963
O	1.40354113	-0.00201161	2.63318963
O	4.24339011	-0.03737402	2.77783838
O	-4.24339011	0.03737402	2.77783838

[1,2] - $[Mo_2V_8O_{28}]^{6-}$ (spin polarised) Symmol = C(2V)

Mo	-1.63856502	-0.00000000	2.35256015
Mo	1.63856502	0.00000000	2.35256015
V	-1.59098236	-0.00000000	-2.31795789
V	1.59098236	0.00000000	-2.31795789
V	-3.10966808	1.59812108	-0.07401272
V	-3.10966808	-1.59812108	-0.07401272
V	-0.00000000	1.61929199	-0.04350074
V	0.00000000	-1.61929199	-0.04350074
V	3.10966808	1.59812108	-0.07401272
V	3.10966808	-1.59812108	-0.07401272
O	-1.40100947	-2.63386018	0.00562778
O	1.40100947	-2.63386018	0.00562778
O	0.00000000	-1.22163013	-1.89309033
O	0.00000000	-1.25232977	1.96938645
O	-1.31986340	-0.00000000	0.05847842
O	1.31986340	0.00000000	0.05847842
O	-2.72256660	-1.35395420	-1.88937333
O	-2.76841891	-1.39092530	1.88356308
O	-4.24032583	-2.77883661	-0.05925918
O	-4.05600743	-0.00000000	-0.00449178
O	2.72256660	-1.35395420	-1.88937333
O	2.76841891	-1.39092530	1.88356308
O	4.05600743	0.00000000	-0.00449178
O	4.24032583	-2.77883661	-0.05925918
O	-0.00000000	1.22163013	-1.89309033
O	-1.55873377	-0.00000000	-3.95350976
O	-2.72256660	1.35395420	-1.88937333
O	1.55873377	0.00000000	-3.95350976
O	2.72256660	1.35395420	-1.88937333
O	-0.00000000	1.25232977	1.96938645
O	2.76841891	1.39092530	1.88356308
O	1.61355266	0.00000000	4.09903423
O	-2.76841891	1.39092530	1.88356308
O	-1.61355266	-0.00000000	4.09903423
O	1.40100947	2.63386018	0.00562778
O	-1.40100947	2.63386018	0.00562778
O	-4.24032583	2.77883661	-0.05925918
O	4.24032583	2.77883661	-0.05925918

[1,2] - $[Mo_2V_8O_{28}]^{6-}$ (closed shell) Symmol = C(2V)

Mo	-1.37863114	0.00000000	2.39467186
Mo	1.37863114	-0.00000000	2.39467186
V	-1.64451668	-0.00000000	-2.31148094
V	1.64451668	-0.00000000	-2.31148094
V	-3.12244994	1.56670996	0.05680187
V	-3.12244994	-1.56670996	0.05680187
V	-0.00000000	1.65443059	-0.18409057
V	0.00000000	-1.65443059	-0.18409057
V	3.12244994	1.56670996	0.05680187
V	3.12244994	-1.56670996	0.05680187
O	-1.38433859	-2.66096443	-0.07291975
O	1.38433859	-2.66096443	-0.07291975
O	0.00000000	-1.22966482	-1.96683715
O	-0.00000000	-1.40696735	1.94159136
O	-1.26010874	-0.00000000	0.03947327
O	1.26010874	0.00000000	0.03947327
O	-2.72604254	-1.34001565	-1.83387928
O	-2.72657968	-1.38232283	1.81276545
O	-4.25180515	-2.74860457	0.02786576
O	-4.09658661	-0.00000000	-0.04843815
O	2.72604254	-1.34001565	-1.83387928
O	2.72657968	-1.38232283	1.81276545
O	4.09658661	-0.00000000	-0.04843815
O	4.25180515	-2.74860457	0.02786576
O	-0.00000000	1.22966482	-1.96683715
O	-1.66880973	-0.00000000	-3.95031754
O	-2.72604254	1.34001565	-1.83387928
O	1.66880973	0.00000000	-3.95031754
O	2.72604254	1.34001565	-1.83387928
O	-0.00000000	1.40696735	1.94159136
O	2.72657968	1.38232283	1.81276545
O	1.68201835	-0.00000000	4.10415975
O	-2.72657968	1.38232283	1.81276545
O	-1.68201835	0.00000000	4.10415975
O	1.38433859	2.66096443	-0.07291975
O	-1.38433859	2.66096443	-0.07291975
O	-4.25180515	2.74860457	0.02786576
O	4.25180515	2.74860457	0.02786576

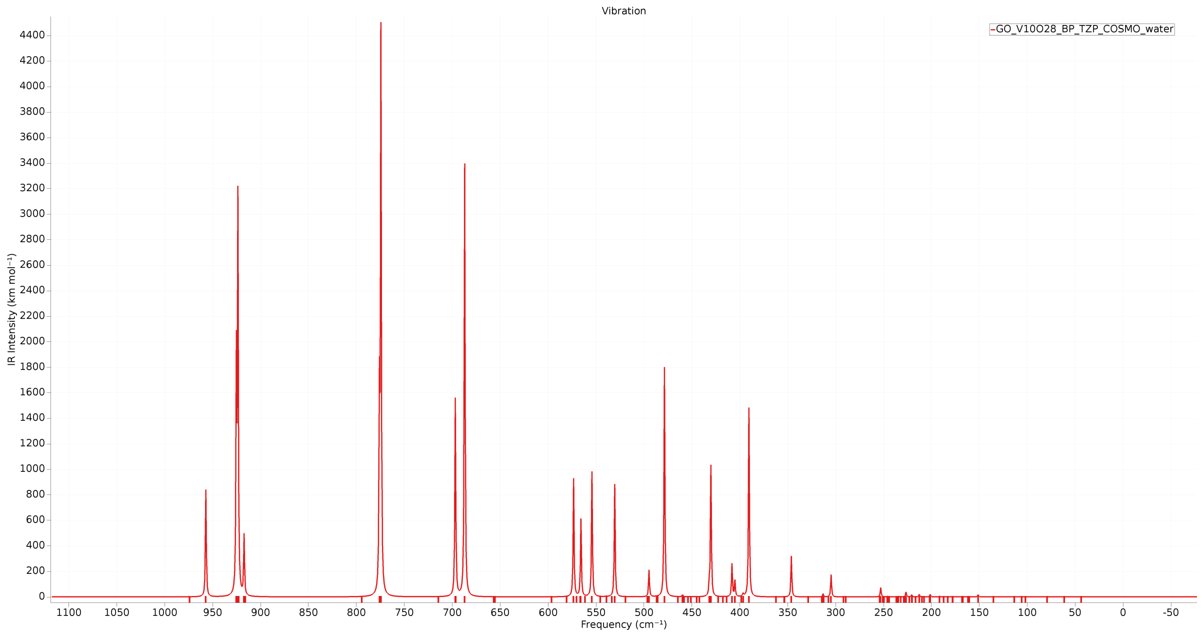

Figure 2: Calculated IR spectrum of $[V_{10}O_{28}]^{6-}$ at BP86/ZORA-scalar/TZP/COSMO-water level.

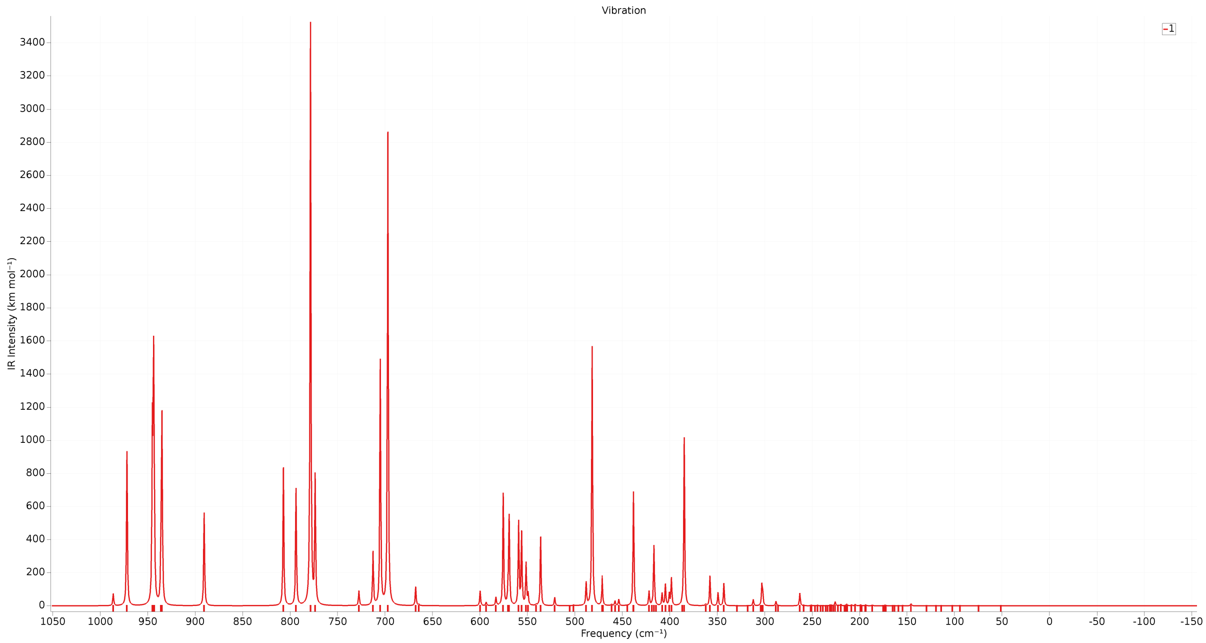

Figure 3: Calculated IR spectrum of [1] $[MoV_9O_{28}]^{5-}$ at BP86/ZORA-scalar/TZP/COSMO-water level.

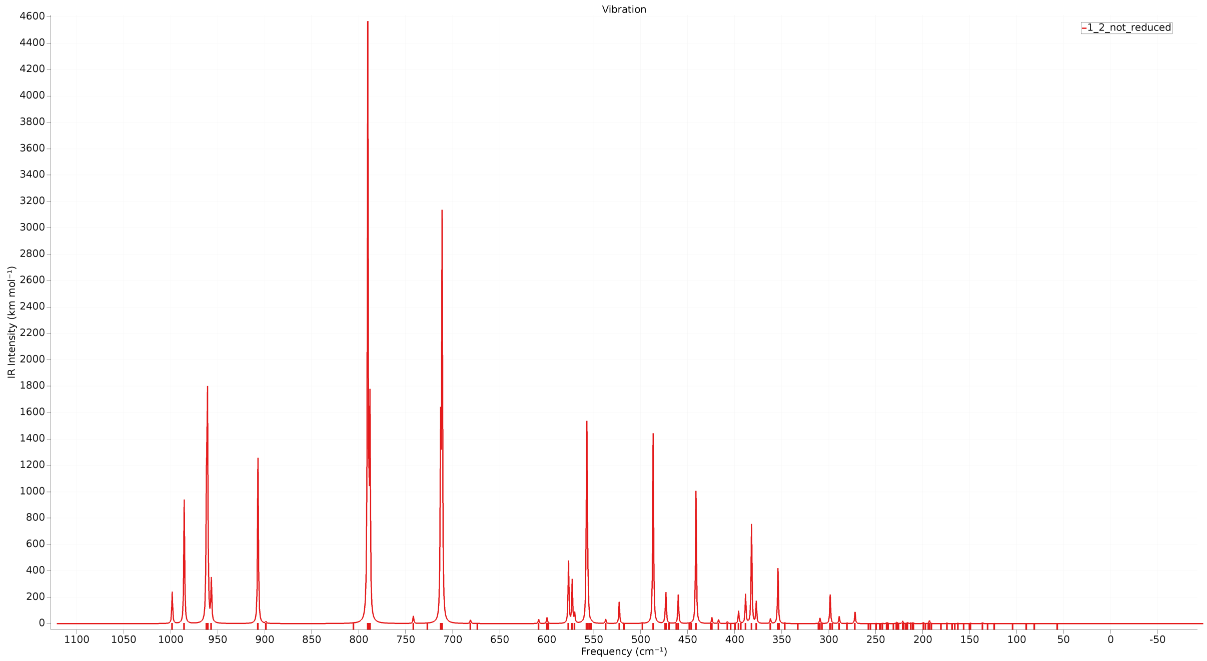

Figure 4: Calculated IR spectrum of [1,2] $[Mo_2V_8O_{28}]^{4-}$ at BP86/ZORA-scalar/TZP/COSMO-water level.

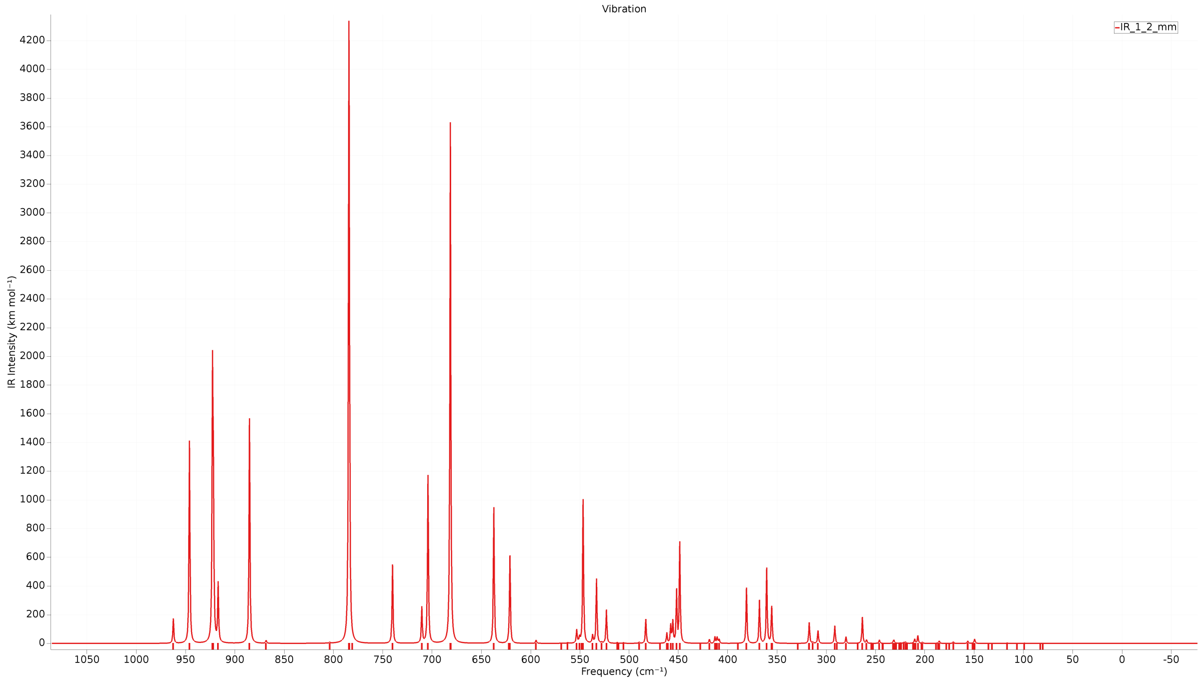

Figure 5: Calculated IR spectrum of [1,2] $[Mo_2V_8O_{28}]^{6-}$ at BP86/ZORA-scalar/TZP/COSMO-water level (closed shell).

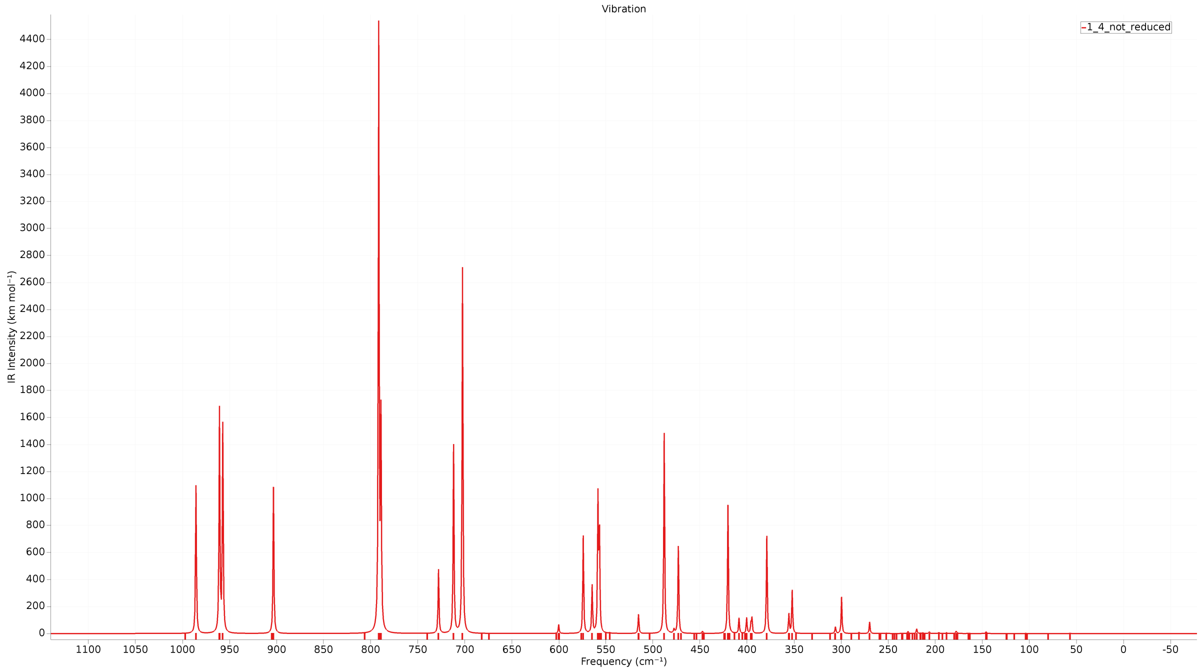

Figure 6: Calculated IR spectrum of [1,4] $[Mo_2V_8O_{28}]^{4-}$ at BP86/ZORA-scalar/TZP/COSMO-water level.

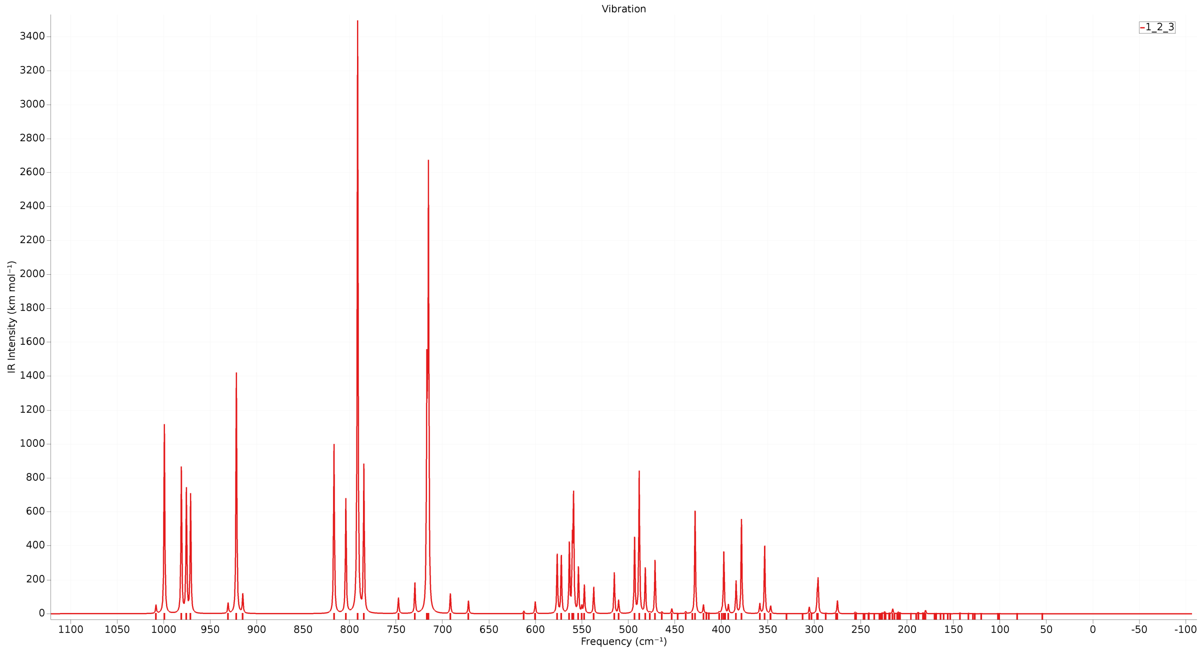

Figure 7: Calculated IR spectrum of [1,2,3] $[Mo_3V_7O_{28}]^{3-}$ at BP86/ZORA-scalar/TZP/COSMO-water level.
